# Cooperativity of steric bulk and H-bonding in coordination sphere engineering: heteroleptic Pd^II^ cages and bowls by design†

**DOI:** 10.1039/d1sc06931d

**Published:** 2022-01-17

**Authors:** Bin Chen, Julian J. Holstein, André Platzek, Laura Schneider, Kai Wu, Guido H. Clever

**Affiliations:** Department of Chemistry and Chemical Biology, TU Dortmund University Otto-Hahn Straße 6 44227 Dortmund Germany guido.clever@tu-dortmund.de; State Key Laboratory of Radiation Medicine and Protection, School for Radiological and Interdisciplinary Sciences (RAD-X), Collaborative Innovation Center of Radiation Medicine of Jiangsu Higher Education Institutions, Soochow University Suzhou 215123 China; Department of Chemistry, University of Cambridge Lensfield Road Cambridge CB2 1EW UK

## Abstract

Recently developed self-assembly strategies allow to rationally reduce the symmetry of metallosupramolecular architectures. In addition, the combination of multiple ligand types without creating compound mixtures has become possible. Among several approaches to realize non-statistical heteroleptic assembly, Coordination Sphere Engineering (CSE) makes use of secondary repulsive or attractive interactions in direct vicinity of the metal nodes. Previously, we used steric congestion to turn dinuclear [Pd_2_L_4_] cages with fourfold symmetry into [Pd_2_L_3_X_2_] (X = solvent, halide) bowl structures. Here, we introduce a new subtype of this strategy based on balancing hydrogen bonding and repulsive interactions between ligands carrying quinoline (L^Qu^) and 1,8-naphthyridine (L^Na^) donors to generate *trans*-[Pd_2_L_2_
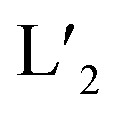
] and [Pd_2_L_3_L′] cages, assisted by templation of encapsulated fullerenes. Combined with steric congestion caused by acridine (L^Ac^) donors, we further report the first example of a heteroleptic [Pd_2_L_2_L′X_2_] bowl. Formation, structure and fullerene binding ability of these metallo-supramolecular hosts were studied by NMR, mass spectrometry and single crystal X-ray diffraction.

## Abstract 2

Coordination Sphere Engineering (CSE) allows non-statistical assembly of heteroleptic supramolecular architectures by fine adjustment of steric and electronic features around square-planar Pd(ii) cations with naphthyridine donors.

## Graphical abstract

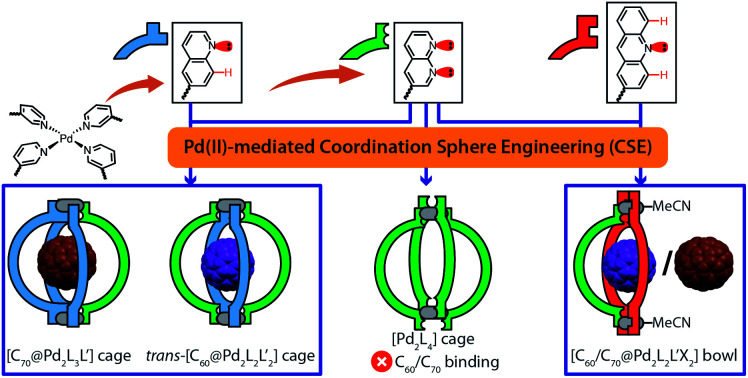

## Introduction

Coordination-driven self-assembly has been accepted as a powerful protocol to efficiently construct highly symmetrical structures with defined cavities that find application in various fields, spanning from molecular recognition, separation techniques, confined catalysis to light harvesting and drug delivery.^1^ In pace with gaining further insight into assembly mechanisms and host–guest properties of such architectures, assembled from metal nodes with predictable coordination geometries and tailor-made organic ligands, an increasing number of researchers has recently begun to study the controlled synthesis of low-symmetry structures composed of more than one type of ligand without creating statistical mixtures.^2^ As nano-sized hosts, such heteroleptic coordination cages promise to achieve advanced applicability, as they allow to introduce a well-chosen combination of functionalities within their cavity. In this respect, this approach is inspired by the complex inner decoration of natural enzyme pockets with a cooperating set of functional groups. Amongst the growing family of low-symmetry discrete metal–organic cages, palladium-mediated assemblies have received most attention owing to their relatively high thermodynamic stability, but sufficient kinetic lability to promote efficient integrative self-sorting.^3^ To cleanly obtain non-symmetric Pd^II^-based assemblies from symmetric bridging ligands, two principal pathways have recently been established: one, termed “assembly-dependent approach” is based on controlling the integrative combination of two or more structurally different ligands within one architecture through effects concerning the overall structure, such as “shape complementary assembly” (SCA) of matching building blocks,^4^ specific ligand–ligand^5^ or ligand–guest secondary interactions^6^ or steric congestion around the ligand backbones that disfavours homoleptic over heteroleptic structures to be formed.^7^ A further principle pathway bases on adjusting the structure and functionalization of the ligands' donor sites right in the vicinity of the bridging metal cations. We coined the term “coordination sphere engineering” (CSE) for this approach. Judicious choices of donor site chemistry, suitable denticity,^8^ charge distribution,^9^ as well as repulsive (*e.g.* steric hindrance),^10^ and attractive interactions (*e.g.* hydrogen bonding)^11^ nearby the coordination center have to be considered within this strategy. Noteworthy in this respect is a recent report on an integrative assembly strategy involving the use of non-symmetrical bridging ligands by Lewis *et al.*^12^

**Fig. 1:**
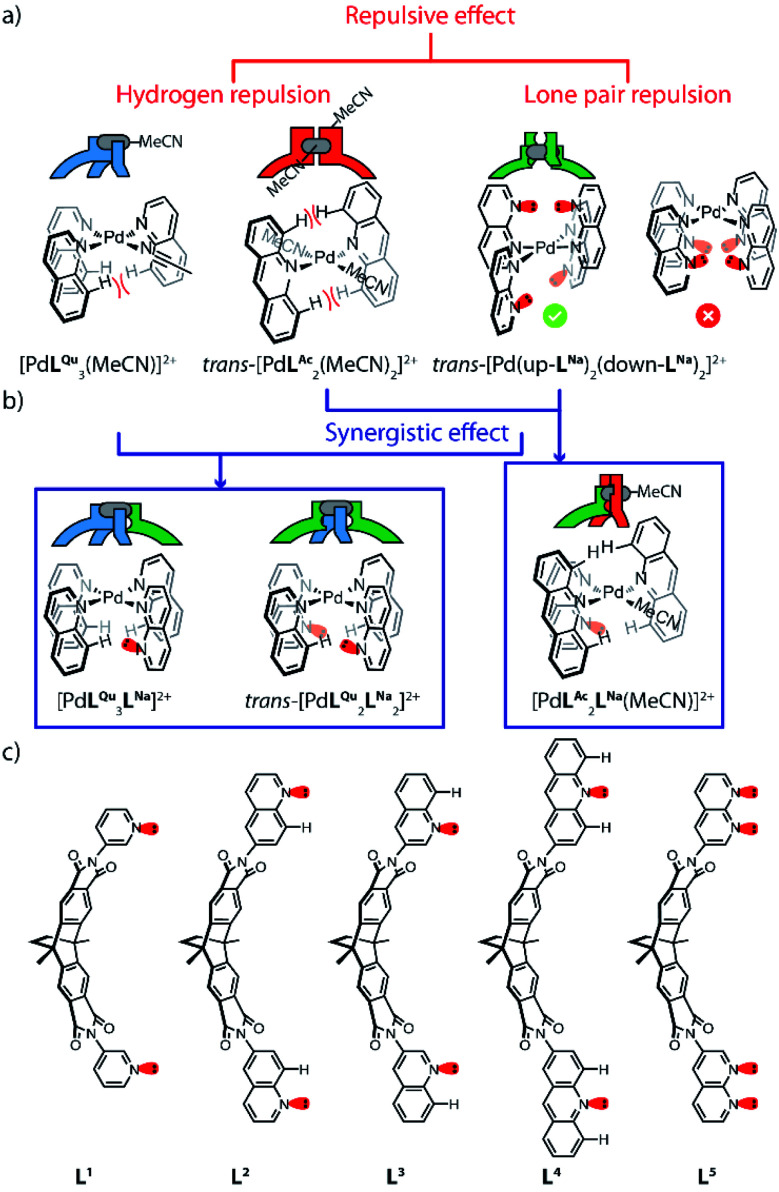
Rational tuning of the square-planar Pd^II^ coordination environment by combining different donor sites: (a) repulsion between quinoline or acridine hydrogen substituents or naphthyridine lone pairs near the congested Pd^II^ center generates unique configurations, *i.e.* [PdL^Qu^_3_(MeCN)]^2+^, *trans*-[PdL^Ac^_2_(MeCN)_2_]^2+^ and *trans*-[Pd(up-L^Na^)_2_(down-L^Na^)_2_]^2+^; (b) synergistic effects between protruding hydrogen atoms and electron-pairs of the donor sites can be exploited to form unique heteroleptic assemblies, *i.e.* [PdL^Qu^_3_L^Na^]^2+^, *trans*-[PdL^Qu^_2_L^Na^_2_]^2+^ and [PdL^Ac^_2_L^Na^(MeCN)]^2+^; (c) chemical structures of studied ligands.

Our group had previously reported a series of bis-monodentate pyridinyl/quinolinyl/acridinyl ligands that assemble with Pd^II^ ions into *D*_4h_-symmetric [Pd_2_L_4_] cages, [Pd_2_L_3_X_2_] (X = solvent, halide) bowls and [Pd_2_L_2_X_4_] rings, respectively.^13^ Key to controlling the metal-to-ligand stoichiometry and overall structure was adjusting the steric congestion around the coordination spheres as induced by inward-pointing hydrogen substituents of the more bulky nitrogen donors quinoline and acridine that come very close upon arrangement around the metal center (Fig. 1a, left).

We further showed that such cages and bowls, when equipped with curved backbones offering a sufficient π-surface contact area, are able to bind fullerenes. Hence, these compounds belong to a growing family of discrete coordination architectures serving as hosts for fullerenes^14^ that are actively studied in recent years in terms of binding capacity,^15^ guest selectivity,^16^ regioselective functionalization,^17^ as well as the controlled release of captured fullerene guests.^18^

Here, we introduce 1,8-naphthyridine as a further donor group for Pd^II^-based assemblies. The same donor has recently been utilized by the Nitschke group in Ag-mediated subcomponent self-assembly, where it was found to show diverse coordination modes with polynuclear Ag clusters.^19^ Initially, we also studied the coordination behavior of Pd^II^ ions with naphthyridine donors posing the question whether dinuclear Pd nodes can be accessed. While this did not happen, we encountered a new structural motif for a [Pd_2_L_4_] cage when four such banana-shaped bifunctional naphthyridine ligands were reacted with Pd^II^ cations, showing an alternative arrangement of neighboring naphthyridine donors with respect to their inner or outer nitrogen donor atoms around the mononuclear coordination centers. Supported by theoretical calculations, the resulting ‘dislocated’ cage structure can be ascribed to the fact that the system avoids repulsive interaction between the nitrogen atoms' electron lone pairs (LPs), that are not involved in metal coordination (Fig. 1a, right). Furthermore, we show that the combination of the new naphthyridine donors with previously reported quinolines and acridines leads to unprecedented heteroleptic structures *via* an interplay between avoidance of repulsive H–H or LP–LP interactions and seemingly attractive interactions between adjacently placed hydrogen and nitrogen atoms (Fig. 1b). Hence, the herein reported donor combinations further enrich the toolbox for creating Pd^II^-assemblies with non-trivial compositions and geometries by the CSE approach.

## Results and discussion

**Fig. 2:**
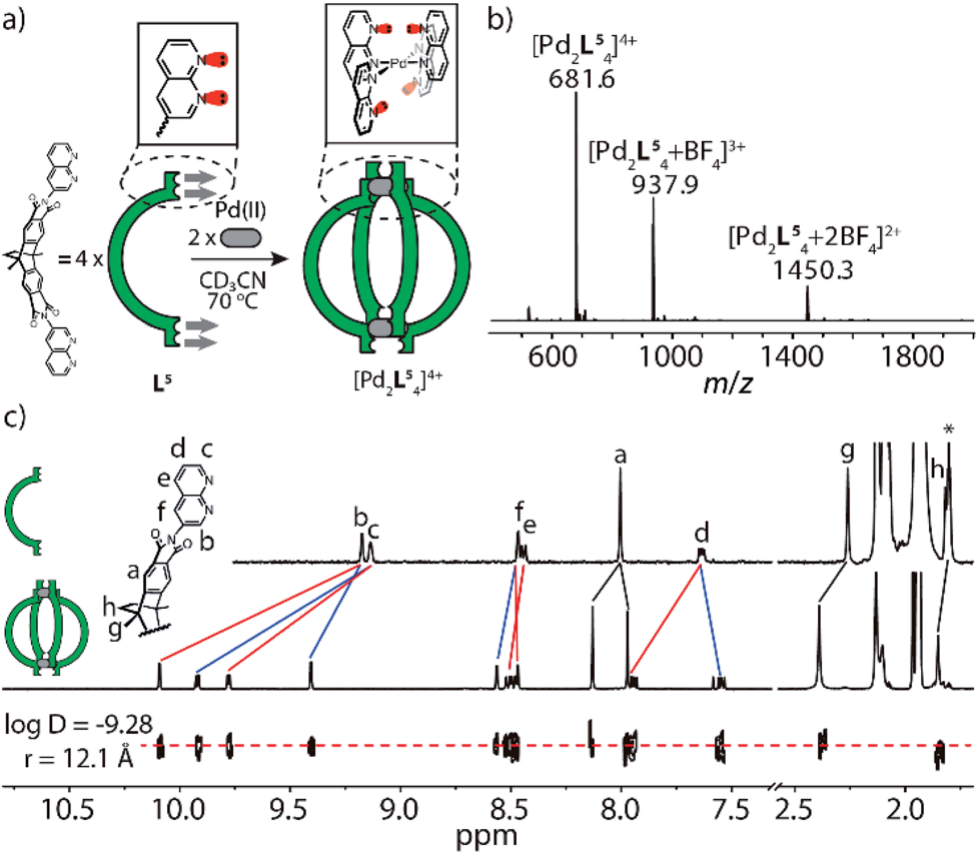
(a) Self-assembly and characterization of homoleptic cage [Pd_2_L^5^_4_] with *trans*-[Pd(up-L^Na^)_2_(down-L^Na^)_2_] ligand arrangement around both coordination sites; (b) high resolution ESI mass spectrum of [Pd_2_L^5^_4_]^4+^ and BF_4_^−^ adducts; (c) ^1^H NMR spectra (600 MHz, 298 K, CD_3_CN) of ligand L^5^ (top), cage [Pd_2_L^5^_4_] (middle, 0.64 mM) and its DOSY trace (bottom).

In order to synthesize new naphthyridine-modified ligand L^5^, the dibenzo-2.2.2-bicyclo-octane backbone that already formed the basis of our previously reported ligands L^1^–L^4^ was equipped with 1,8-naphthyridine donors according to standard condensation procedures (Fig. 1c). Ligand L^5^ was then reacted with palladium source [Pd(MeCN)_4_](BF_4_)_2_ in an NMR titration experiment, suggesting that a thermodynamic product forms with ligand/Pd ratio of 0.50, as no further change of proton signals was observed after addition of Pd^II^ cations beyond this stoichiometry (Fig. 2c and S4†). Further, this product was identified as [Pd_2_L^5^_4_]^4+^ species by ESI mass spectrometry (Fig. 2b). Therefore, it could be inferred that charge repulsion prevents two Pd^II^ cations coordinating in close proximity to both nitrogen atoms of the same naphthyridine donor and thus hampers the generation of tetranuclear [Pd_4_L^5^_4_]^8+^ species (unlike what Nitschke *et al.* observed when using Ag^I^ cations).^19^ In contrast to the larger family of previously encountered [Pd_2_L_4_]-type cages with *D*_4h_-symmetry, further NMR analysis revealed that the proton signals of the naphthyridine moieties (H_b_–H_f_) as well as backbone proton H_a_ split into two sets of peaks with the same intensity, whereas no splitting was observed for the two single peaks assigned to methyl and methylene protons located in the center of the backbones (H_g_, H_h_). All proton signals belong to the same molecular diffusion coefficient (*D* = 5.3 × 10^−10^ m^2^ s^−1^) in the DOSY spectrum (Fig. 2c), indicating that the naphthyridine donors adopt two distinct coordination environments in a single [Pd_2_L^5^_4_] assembly. Diffusion of isopropyl ether into an acetonitrile solution of [Pd_2_L^5^_4_] containing SbF_6_^−^ counterions afforded crystals suitable for X-ray analysis, which helped to shed light on the unprecedented structure of cage [Pd_2_L^5^_4_] featuring an alternative, dislocated arrangement of the four ligands (Fig. 5a and S41†). Here, each Pd^II^-coordination site shows a *trans*-[Pd(up-L^Na^)_2_(down-L^Na^)_2_] donor arrangement as shown in Fig. 1a and each ligand is involved in the ‘up’ coordination mode on one metal center and the ‘down’ mode on the other, rendering the whole structure to show an idealized *D*_2d_ symmetry (distorted by propeller arrangement of the donors and packing effects). The solid-state structure of cage [Pd_2_L^5^_4_] is not only fully consistent with experimental results (NMR/MS) but also the energetically favorable geometry (96.4 kJ mol^−1^ lower) relative to an isomer with ‘all-up’ donor arrangement on one side and ‘all-down’ coordination mode on the other side (which could have also explained the observed NMR splitting pattern), as reflected by DFT calculations (Fig. S43†). We propose that this particular *trans*-[Pd(up-L^Na^)_2_(down-L^Na^)_2_] configuration allows to minimize repulsive interactions between the non-coordinating naphthyridine lone-pairs close to the congested coordination centers. It is worth mentioning that cage [Pd_2_L^5^_4_], compared to [Pd_2_L^1^_4_]^13*a*,*b*^ based on the same backbone, has a reduced volume of its internal cavity by the dislocated ligand arrangement, and is thus deprived of any fullerene binding ability among the series of closely related [Pd_2_L_4_] cages.^13*b*^

Inspired by the prevalence of hydrogen-bonding interactions in supramolecular assembly, we envisioned that the repulsive effects between neighboring hydrogen substituents/electron pairs in the discussed assemblies based on quinoline/acridine and naphthyridine donors, respectively, could be turned into attractive secondary interactions (C_arom_H⋯N_naph_ hydrogen bonds) that should promote the exclusive formation of heteroleptic cages assembled by a combination of these ligands.

**Fig. 3:**
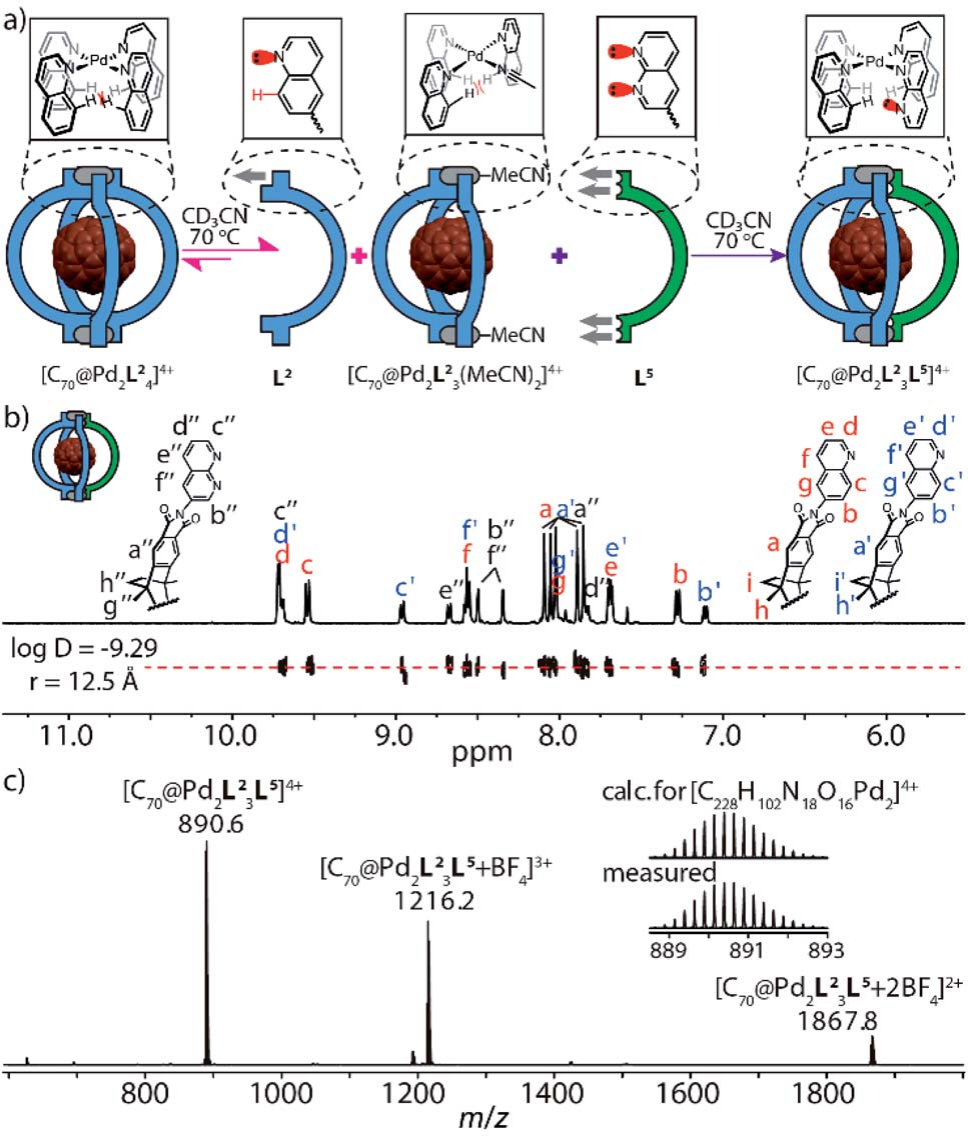
Self-assembly and characterization of heteroleptic cage [C_70_@Pd_2_L^2^_3_L^5^]: (a) when bowl [C_70_@Pd_2_L^2^_3_(MeCN)_2_] reacts with ligand L^2^ or L^5^ in a 1 : 1 ratio at 70 °C, the former ligand only yields minor amounts of cage [Pd_2_L_4_] while the latter leads to quantitative conversion; (b) ^1^H NMR and DOSY spectra (600 MHz, 298 K, CD_3_CN) of cage [C_70_@Pd_2_L^2^_3_L^5^] (0.64 mM); (c) high-resolution ESI mass spectrum of [C_70_@Pd_2_L^2^_3_L^5^] and BF_4_^−^ adducts.

In this line, the treatment of a solution of C_70_-filled molecular bowl [C_70_@Pd_2_L^2^_3_(MeCN)_2_], based on quinoline ligand L^2^, with one equivalent of naphthyridine ligand L^5^ indeed yielded quantitative formation of heteroleptic cage [C_70_@Pd_2_L^2^_3_L^5^] with a rarely observed 3 : 1 ligand stoichiometry in [Pd_2_L_4_] cages (Fig. 3a, right, and Fig. 5c).^20^ It is worth comparing this outcome to the treatment of bowl [C_70_@Pd_2_L^2^_3_(MeCN)_2_] with a fourth equivalent of ligand L^2^, where an equilibrium is reached at a bowl/cage ratio of 4 : 1 (Fig. 3a, left),^13*a*,*b*^ so far from the quantitative situation reached with ligand L^5^ serving as fourth assembly partner. The exclusive formation of [C_70_@Pd_2_L^2^_3_L^5^], on the other hand, was supported by NMR, DOSY and high-resolution ESI mass spectrometry (Fig. 3b and c). Hence, we show that a rather subtle modification of the donor group in vicinity of the square-planar Pd^II^ coordination sphere, *i.e.* substitution of a CH unit by a nitrogen atom, significantly changes the fidelity of installing a fourth ligand on a bowl structure, which we attribute to favorable secondary electrostatic attraction between the quinoline and naphthyridine donor groups.

**Fig. 4:**
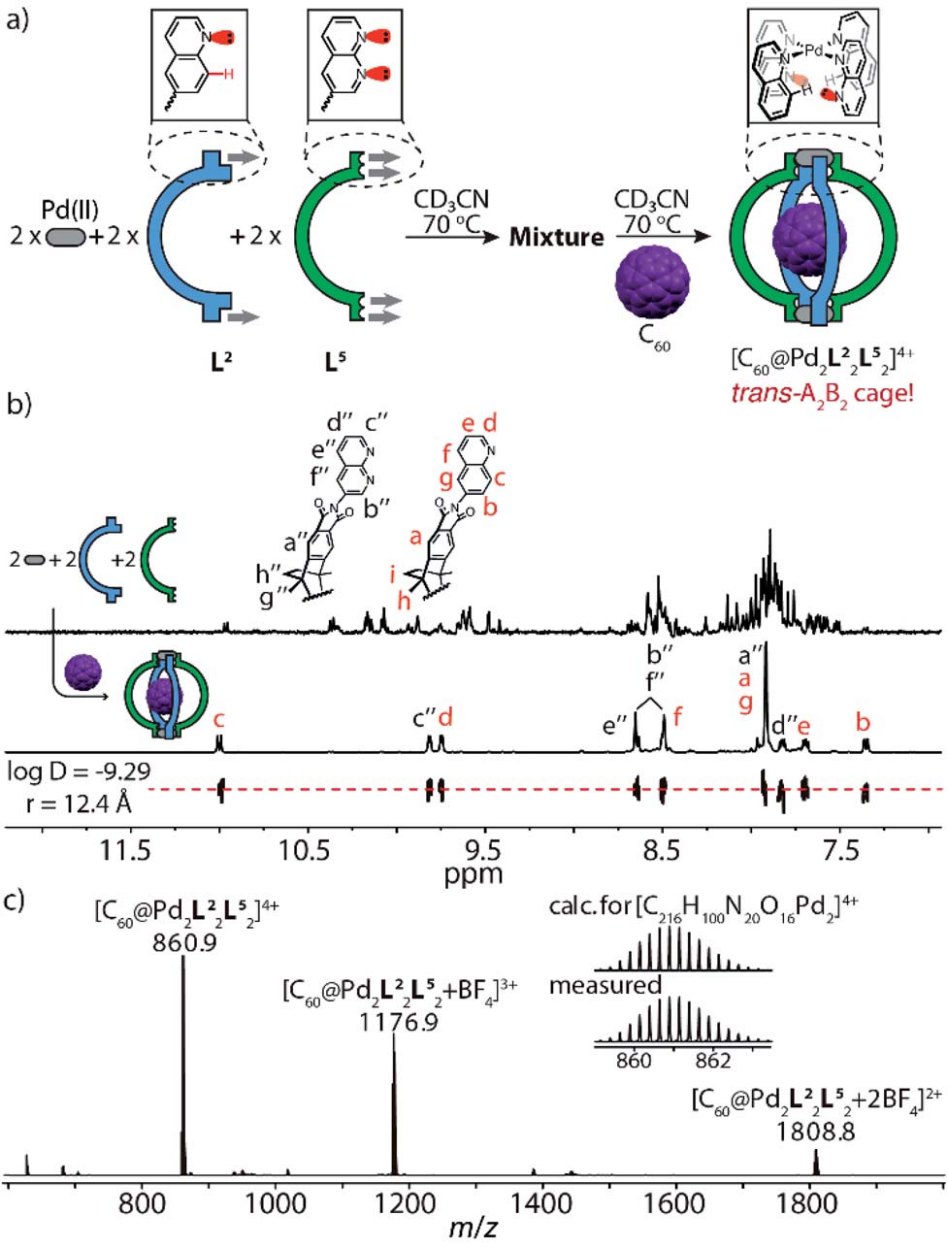
Self-assembly and characterization of heteroleptic cage [C_60_@Pd_2_L^2^_2_L^5^_2_]: (a) ligands L^2^ and L^5^ react with Pd^II^ cations in a 1 : 1 : 1 ratio at 70 °C to give a convoluted mixture, followed by the addition of C_60_, leading to social self-sorting to give *trans*-[C_60_@Pd_2_L^2^_2_L^5^_2_]; (b) ^1^H NMR spectra (600 MHz, 298 K, CD_3_CN) of the reaction mixture of Pd^II^/L^2^/L^5^ in a 1 : 1 : 1 ratio and cage [C_60_@Pd_2_L^2^_2_L^5^_2_] (0.64 mM, bottom: DOSY trace); (c) high-resolution ESI mass spectrum of [C_60_@Pd_2_L^2^_2_L^5^_2_] and BF_4_^−^ adducts.

This finding emboldened us to further explore the assembly of ligands L^2^ and L^5^ with Pd^II^ cations under strict stoichiometric control. An experiment to screen the Pd^II^-mediated assembly starting from different ligand ratios (Fig. S34†) suggested that in the absence of fullerene guests, the attractive interaction between the complementary donor sites is insufficient to cleanly form the expected heteroleptic cages. Yet, fullerene guests, acting as templates,^6*c*^ are able to trigger the generation of two heteroleptic cages with high fidelity (Fig. S35 and S36†): one is the A_3_B-type [C_70_@Pd_2_L^2^_3_L^5^] system as mentioned above, another is the A_2_B_2_-type cage [C_60_@Pd_2_L^2^_2_L^5^_2_] (Fig. 4), as detailed in the following: heating a mixture of Pd^II^/L^2^/L^5^ in 1 : 1 : 1 ratio affords a convoluted mixture of multiple species as indicated by a large number of proton signals in the respective NMR spectrum (Fig. 4b, top). Pleasingly, the spectrum significantly simplifies upon the addition of powdered C_60_ into the mixture, followed by stirring at elevated temperature (Fig. 4a and b, bottom). The question arises whether cage [C_60_@Pd_2_L^2^_2_L^5^_2_] adopts a *cis*- or *trans*-configuration. NMR delivers the answer as all proton signals of cage [C_60_@Pd_2_L^2^_2_L^5^_2_] assigned by 2D NMR spectra belong to a single species (with common DOSY-derived diffusion coefficient of 5.1 × 10^−10^ m^2^ s^−1^) and protons of both ligands found on the left and right sides of the ribbon-shaped backbones (H_a_, H_h_, H_i_ for L^2^; H_a′′_, H_g′′_, H_h′′_ for L^5^) do not give rise to any signal splitting (Fig. S16†), thus pointing to a relatively high *trans*-configured symmetry of the overall cage. Further, the *trans*-isomer was found to be 13.6 kJ mol^−1^ lower in energy than a tentative *cis*-geometry, as determined by DFT calculation (Fig. S44†). In a more general way, the favorable combination of the two donor types in *trans*-arrangement around a Pd^II^ center (in contrast to a *cis*-arrangement) as well as not observing any structures composed of three or even four naphthyridine ligands arranged around the same metal (in a non-up/down situation as in [Pd_2_L^5^_4_]) was further supported by a DFT study comparing a series of tentative mononuclear model complexes (Fig. S45†).

**Fig. 5:**
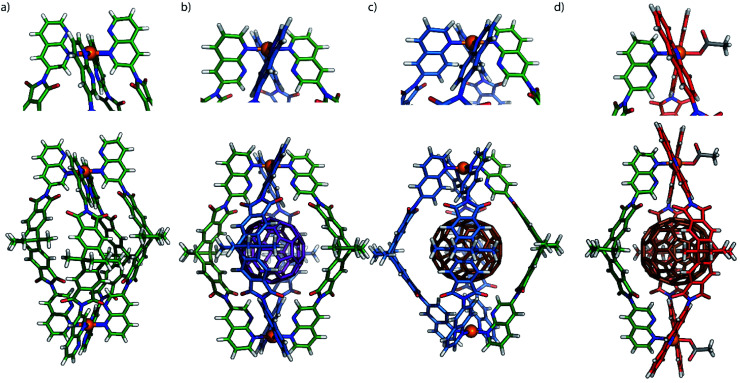
(a) X-ray structure of [Pd_2_L^5^_4_]; (b) gas-phase B3LYP/LANL2DZ DFT-optimized models of *trans*-[C_60_@Pd_2_L^2^_2_L^5^_2_] and (c) [C_70_@Pd_2_L^2^_3_L^5^]; (d) X-ray structure of [C_70_@Pd_2_L^4^_2_L^5^(OAc)_2_]. Colours: L^2^: blue, L^4^: red, L^5^: green, C_60_: purple, C_70_: brown. Anions and solvents molecules omitted. For X-ray diffraction and DFT calculation details see ESI.†

It is noteworthy that the quinoline proton signal H_c_ of cage [C_60_@Pd_2_L^2^_2_L^5^_2_] was found at 11.0 ppm in the NMR spectrum and thus undergoes a striking downfield-shift by about 2 ppm compared with its position in the NMR spectra of other species (Tab. S1†). This protruding hydrogen atom H_c_ is thus observed to be de-shielded by the adjacent lone pairs of the neighboring naphthyridine ligands within the confined space next to the Pd^II^-coordination sphere, direct evidence of a secondary electrostatic attraction between the quinoline and naphthyridine donors. More intriguingly, C_60_-binding experiments with homoleptic cages [Pd_2_L^2^_4_] and [Pd_2_L^5^_4_] show that neither can accommodate a fullerene in their internal cavities,^13*b*^ in contrast to the product of their integrative 1 : 1 assembly upon mixing, which can bind one C_60_ to give heteroleptic cage *trans*-[C_60_@Pd_2_L^2^_2_L^5^_2_] (Fig. 5b). This further highlights the synergistic effect between the electronically complementary quinoline and naphthyridine donor groups on ligands L^2^ and L^5^.

**Fig. 6:**
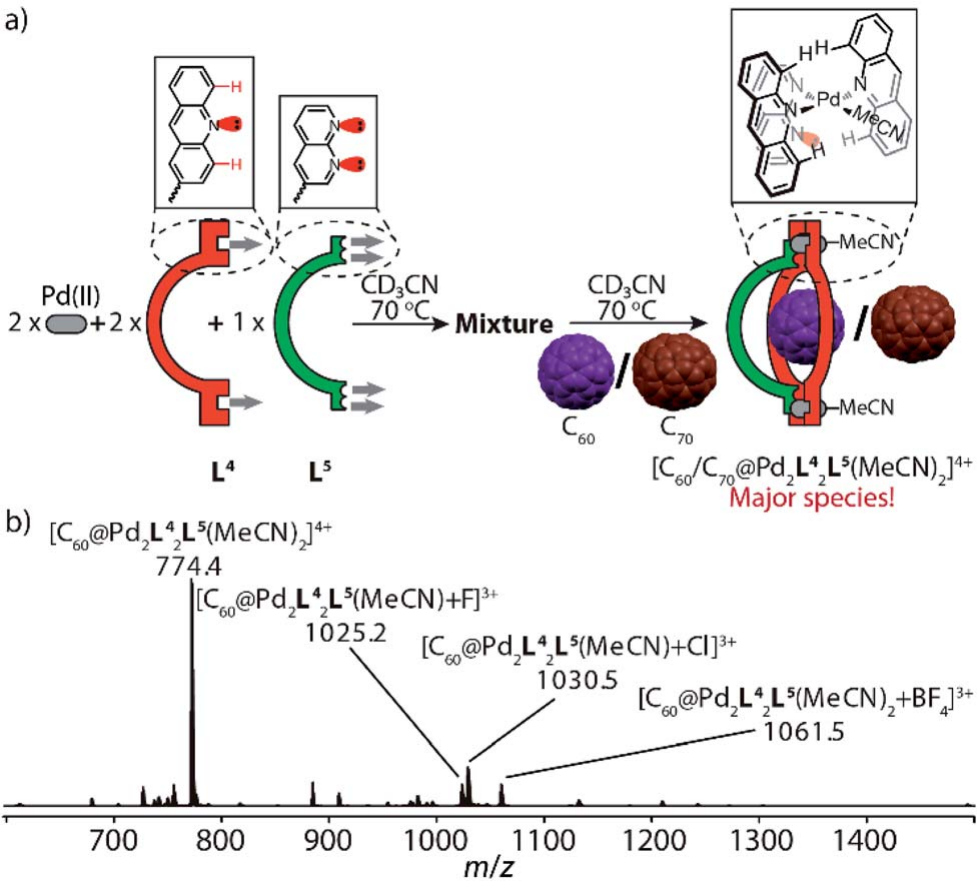
Self-assembly of fullerene-containing heteroleptic bowl [C_60_/C_70_@Pd_2_L^4^_2_L^5^(MeCN)_2_]: (a) ligands L^4^ and L^5^ react with Pd^II^ cations in a 2 : 1 : 2 ratio at 70 °C to give a convoluted mixture, followed by the addition of C_60_ or C_70_ to produce heteroleptic bowl [C_60_/C_70_@Pd_2_L^2^_2_L^5^_2_] as major species; (b) high-resolution ESI mass spectrum of [C_60_@Pd_2_L^4^_2_L^5^(MeCN)_2_] and its anion adducts.

Next, we expanded this strategy to probe heteroleptic cage/bowl formation based on acridine ligand L^4^ and naphthyridine L^5^ in a similar way. Screening different ratios of Pd^II^ cations, ligands L^4^ and L^5^ in presence of fullerene guests C_60_ or C_70_ yielded that a 2 : 2 : 1 mixture of Pd/L^4^/L^5^ heated at 70 °C produces fullerene-filled heteroleptic bowls [C_60_@Pd_2_L^4^_2_L^5^(MeCN)_2_] and [C_70_@Pd_2_L^4^_2_L^5^(MeCN)_2_] as major species, respectively (Fig. 6b, S38 and S39†). The succinct proton signals in their NMR spectra (Fig. S22 and S28†) confirm that naphthyridine ligand L^5^ is capable of bridging acridine-based molecular ring [Pd_2_L^4^_2_(MeCN)_4_] to give heteroleptic bowls, which is again facilitated by the templating effect of the fullerene guests (Fig. 6a). Red needle-shaped crystals were obtained by slow vapor diffusion of benzene into a CD_3_CN solution of [C_70_@Pd_2_L^4^_2_L^5^(MeCN)_2_](BF_4_)_4_. X-ray analysis shows that the loosely coordinated acetonitrile molecules were substituted by acetate ions (probably solvent contaminants) in the crystal structure (Fig. 5d). Careful inspection of the [PdL^Ac^_2_L^Na^(OAc)]^+^ coordination nodes shows that the shortest distance between the hydrogen atom (H_c_) in the 4-acridinyl-position and the uncoordinated nitrogen atom of the naphthyridine donor is only 2.56 Å, which explains the downfield-shifted signal of proton H_c_ (at 10.4 ppm) in the NMR spectrum of [C_70_@Pd_2_L^4^_2_L^5^(MeCN)_2_] (Fig. S28†).

## Conclusions

In summary, 1,8-naphthyridine enriches the toolbox for the coordination sphere engineering (CSE) approach as alternative donor for Pd^II^ cations as it allows to exploit both lone pair repulsion effects between its uncoordinated nitrogen atoms as well as attractive interactions with hydrogen substituents of matching quinoline or acridine donors in direct neighborhood. Hence, while homoleptic [Pd_2_L_4_] assemblies of naphthyridine ligand L^5^ feature a unique dislocated geometry due to repulsive lone pair interactions, combination of this ligand with previously reported ligand derivatives allows for the fullerene-templated generation of unprecedented heteroleptic cage and bowl structures. The herein described donor-induced synergistic effects form the structural basis for the controlled, non-statistical synthesis of a new generation of sophisticated fullerene-containing assemblies with diverse functionalities, *e.g.* chromophores or redox-moieties, implemented in differentiable ligand backbones, allowing to develop nano devices and materials for light-harvesting, catalytic and electronic applications.

## Data availability

Crystallographic data for compounds L^5^, [Pd_2_L^5^_4_](SbF_6_)_4_ and [C_70_@Pd_2_L^4^_2_L^5^(OAc)_2_](BF_4_)_2_(C_6_H_6_)_2_ has been deposited at the CCDC database under CCDC numbers 1997307–1997309 and can be obtained from The Cambridge Crystallographic Data Centre *via* http://www.ccdc.cam.ac.uk/data_request/cif. Further analytical data is reported in the ESI to this article.

## Author contributions

B. Chen and G. H. Clever conceived and designed the study. B. Chen performed the synthesis and characterization of the materials. J. J. Holstein, A. Platzek, L. Schneider, and K. Wu assisted in structural characterization (X-ray, NMR, and MS analyses). B. Chen wrote the original draft and G. H. Clever performed computational studies, reviewed and edited the paper.

## Conflicts of interest

There are no conflicts to declare.

## Supplementary Material

SC-013-D1SC06931D-s001

SC-013-D1SC06931D-s002
