# Supplementary material for: Cooperativity of steric bulk and H-bonding in coordination sphere engineering: heteroleptic PdII cages and bowls by design
Source: Chem Sci. 2022 Jan 17;13(6):1829–34. doi: 10.1039/d1sc06931d (PMC8826863; doi:10.1039/d1sc06931d)
Supplement: SC-013-D1SC06931D-s001 [file SC-013-D1SC06931D-s001.pdf]

Electronic Supplementary Material (ESI) for Chemical Science.
This journal is © The Royal Society of Chemistry 2022

Contents:

1 Materials and methods **2**

1.1 Materials *2*

1.2 Purification and analytical techniques *2*

2 Synthesis of ligand L^5 **3**

3 Formation and characterization of metallosupramolecular assemblies **4**

3.1 Titration of ligand L^5 with $[Pd(MeCN)_4](BF_4)_2$ *4*

3.2 Formation and characterization of homoleptic cage $[Pd_2L^5_4]^{4+}$ *5*

3.3 Formation and characterization of heteroleptic cage $[C_{70}@Pd_2L^2_3L^5]^{4+}$ *8*

3.4 Formation and characterization of heteroleptic cage $[C_{60}@Pd_2L^2_2L^5_2]^{4+}$ *11*

3.5 Formation and characterization of heteroleptic cage $[C_{60}@Pd_2L^4_2L^5(MeCN)_2]^{4+}$ *14*

3.6 Formation and characterization of heteroleptic cage $[C_{70}@Pd_2L^4_2L^5(MeCN)_2]^{4+}$ *18*

4 Fullerene binding investigation of cage $[Pd_2L^5_4]^{4+}$ **20**

5 Reactions of mixed ligand systems **21**

5.1 Reactions of $[Pd(MeCN)_4](BF_4)_2$, ligands L^2 and L^5 *21*

5.2 Reactions between $[Pd(MeCN)_4](BF_4)_2$, ligands L^4 and L^5 *24*

6 X-Ray data **26**

6.1 General methods *26*

6.2 Crystal structure of ligand L^5 *27*

6.3 Crystal structure of $[Pd_2L^5_4](SbF_6)_4$ *28*

6.4 Crystal structure of $[C_{70}@Pd_2L^4_2L^5(OAc)_2](BF_4)_2(C_6H_6)_2$ *31*

7 Computational studies **32**

7.1 Comparison of the DFT minimized energies of A-type $[Pd_2L^5_4]^{4+}$ and B-type $[Pd_2L^5_4]^{4+}$ *33*

7.2 Comparison of the DFT minimized energies of cis-$[C_{60}@Pd_2L^2_2L^5_2]^{4+}$ and trans-$[C_{60}@Pd_2L^2_2L^5_2]^{4+}$ *33*

7.3 Comparison of ligand combinations in mononuclear model complexes *34*

8 References **35**

1 Materials and methods

1.1 Materials

All chemicals were obtained from commercial sources and used without further purification. Fullerenes C_{60} and C_{70} were purchased from ABCR with a purity of 99.95% and Sigma-Aldrich with a purity of 98%, respectively.

Syntheses and characterization of ligands **L²** and **L⁴** as well as their self-assembled cages, bowls and rings, i.e. $[Pd_2\mathbf{L^2}_3(MeCN)_2]^{4+}$, $[Pd_2\mathbf{L^2}_4]^{4+}$, $[C_{60}@Pd_2\mathbf{L^2}_3(MeCN)_2]^{4+}$, $[C_{70}@Pd_2\mathbf{L^2}_3(MeCN)_2]^{4+}$, $[C_{70}@Pd_2\mathbf{L^2}_4]^{4+}$ and $[Pd_2\mathbf{L^4}_2(MeCN)_4]^{4+}$ have been reported previously.[1-2]

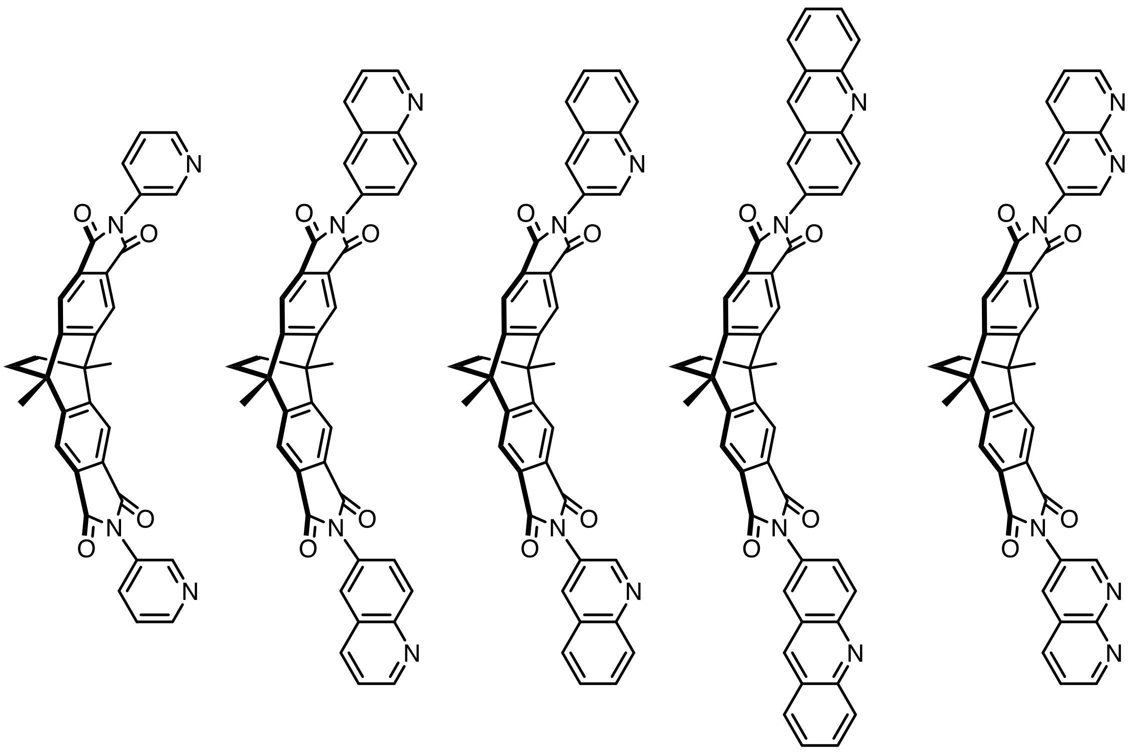

L¹ **L²** **L³** **L⁴** **L⁵**

Figure S1 Chemical structures of this series of ligands.

1.2 Purification and analytical techniques

Gel permeation chromatography (GPC) purification of ligands was performed on a JASCO LC-9210 II NEXT running with $CHCl_3$ (HPLC grade) containing 0.5% (v/v) triethylamine. Flash chromatography was performed on a Biotage Isolera One fraction collector with Biotage SNAP Ultra columns. NMR measurements were all conducted at 298 K on Avance-500 neo and Avance-600 instruments from Bruker and an INOVA 500 MHz machine from Varian. Chemical shifts for ^{1}H and ^{13}C are reported in ppm on the δ scale; ^{1}H and ^{13}C signals were referenced to the residual solvent peak: acetonitrile (1.94 ppm, 1.32 ppm); chloroform (7.26 ppm, 77.16 ppm). The following abbreviations are used to describe signal multiplicity for ^{1}H NMR spectra: s: singlet, d: doublet, t: triplet, dd: doublet of doublets; dt: doublet of triplets; m: multiplet, br: broad. All

proton signals of supramolecular compounds were assigned with the aid of 2D NMR spectra. High resolution electrospray ionization mass spectrometry (ESI HRMS) was performed on Bruker Apex IV ESI-FTICR and Bruker ESI timsTOF and Bruker compact mass spectrometers.

2 Synthesis of ligand L5

Ligand **L5** was prepared from reported bis-anhydride (9,10-dimethyl-9,10-dihydro-9,10-ethanoanthracene-2,3,6,7-dianhydride)[3] and powdered 3-amino-1,8-naphthyridine under nitrogen atmosphere as described below.

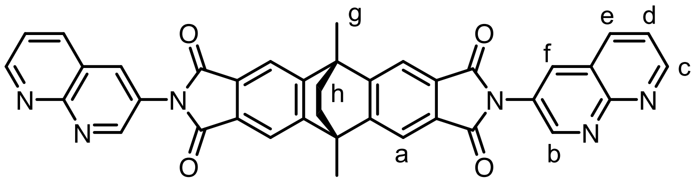

L5

Under a nitrogen atmosphere, ligand **L5** was prepared from reported bis-anhydride (9,10-dimethyl-9,10-dihydro-9,10-ethanoanthracene-2,3,6,7-dianhydride) (74.8 mg, 0.20 mmol, 1 eq.) and powdered 3-amino-1,8-naphthyridine (145.2 mg, 1.00 mmol, 5 eq.) by heating the mixture of solids without solvent in a preheated oil bath to 170 °C for 10 min. After the black melt cooled to room temperature, it was taken up into 10 mL chloroform, sonificated and the suspension was immediately subjected to flash column chromatography on silica gel ($CHCl_3$: MeOH = 50 : 1) to give the crude product. This was further purified via recycling gel permeation chromatography and the solvent was removed under reduced pressure to yield the desired product as a white powder (48.3 mg, 38 %).

^{1}H NMR (600 M, 298 K, $CDCl_3$): δ (ppm) = 9.31 (d, J = 2.7 Hz, 2H), 9.20 (dd, J = 4.3, 1.9 Hz, 2H), 8.39 (d, J = 2.7 Hz, 2H), 8.32 (dd, J = 8.2, 2.0 Hz, 2H), 8.01 (s, 4H), 7.60 (dd, J = 8.2, 4.2 Hz, 2H), 2.25 (s, 6H), 1.83 (s, 4H).

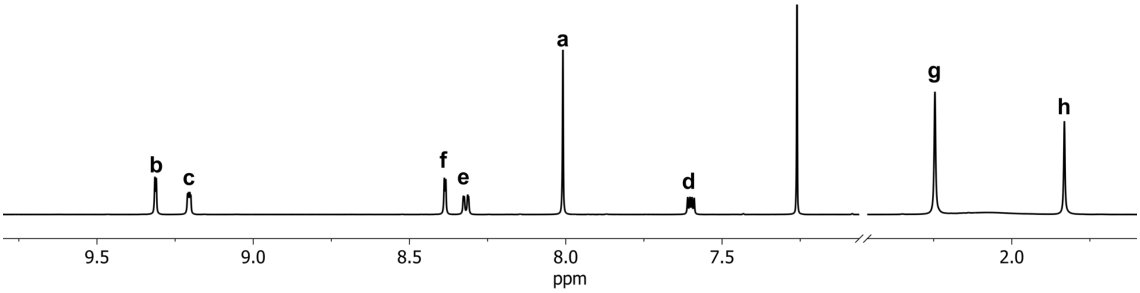

Figure S2 ^{1}H NMR spectrum (600 MHz, 298 K, $CDCl_3$) of **L5**.

^{13}C NMR (151 MHz, 298 K, $CDCl_3$): δ (ppm) = 166.96, 154.22, 153.95, 153.03, 151.24, 137.91, 132.97, 130.06, 126.95, 122.95, 122.51, 117.12, 44.46, 34.97, 18.86.

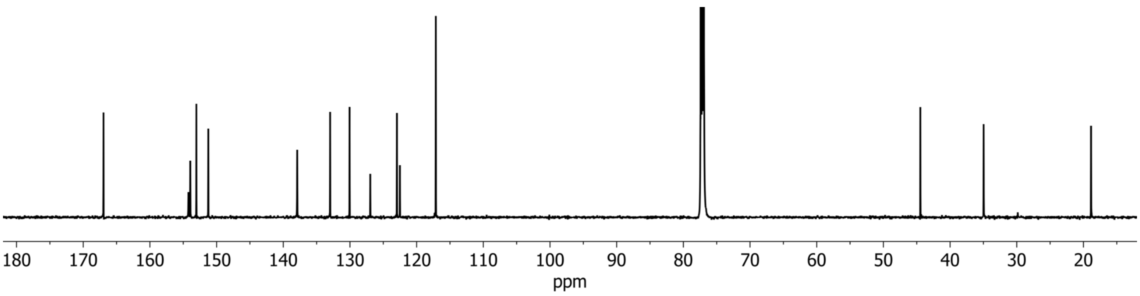

Figure S3 ^{13}C NMR spectrum (151 MHz, 298 K, $CDCl_3$) of **L5**.

ESI HRMS ($C_{38}H_{24}N_6O_4$): $[M + H]^+$ calcd. for $C_{38}H_{25}N_6O_4$ 629.1932; found 629.1910; $[M + 2H]^{2+}$ calcd. for $C_{38}H_{26}N_6O_4$ 315.1002; found 315.0995.

3 Formation and characterization of metallosupramolecular assemblies

3.1 Titration of ligand L^5 with $[Pd(MeCN)_4](BF_4)_2$

The sparingly soluble ligand **L5** (1.1 mg, 1.80 µmol, 1 eq.) in CD_3CN solution (642 µL) was titrated with a concentrated CD_3CN solution of $[Pd(MeCN)_4](BF_4)_2$ (15 mM). Upon each addition of 0.25 eq. $[Pd(MeCN)_4](BF_4)_2$ (15 mM, 30.0 µL, 0.45 µmol), the solution was shaken and heated at 70 °C for 1 d before acquiring the NMR spectrum, which allowed equilibrium to be reached.

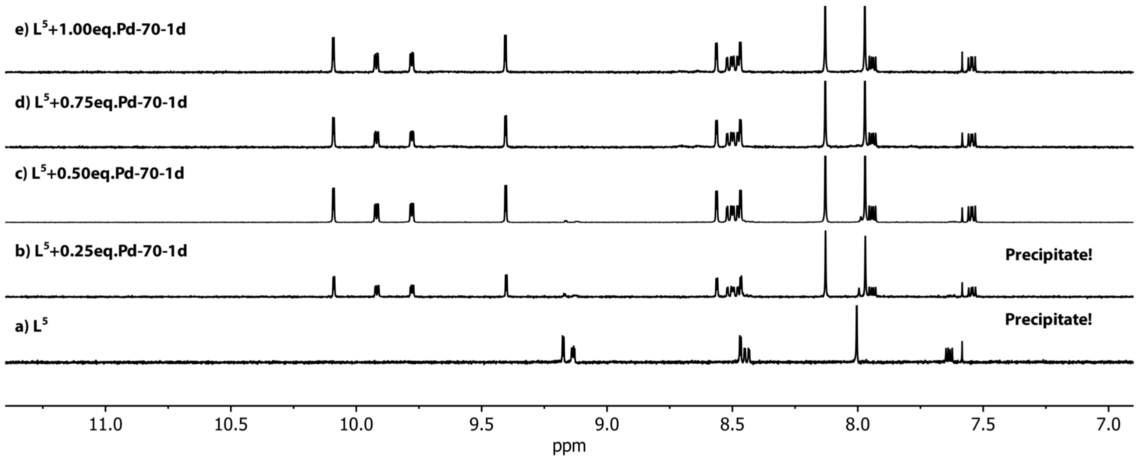

Figure S4 ^{1}H NMR titration (500 MHz, 298 K, CD_3CN) of **L5** with $[Pd(MeCN)_4](BF_4)_2$. Upon addition of 0.5 eq. $[Pd(MeCN)_4](BF_4)_2$, the solution became clear without precipitate remaining in the bottom. No further chemical shifting of proton signals were observed in spectra after continuous addition of Pd^{II} cations.

3.2 Formation and characterization of homoleptic cage $[Pd_2L^5_4]^{4+}$

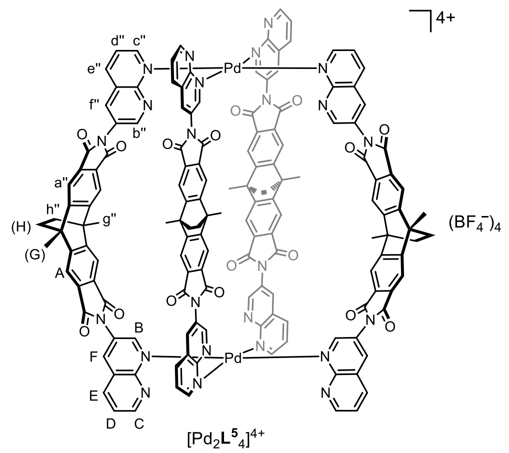

A solution of $[Pd(MeCN)_4](BF_4)_2$ (166.0 µL, 15 mM/CD_3CN, 2.49 µmol, 1 eq.) was combined with ligand **L⁵** (3.1 mg, 4.98 µmol, 2 eq.) in CD_3CN (1778 µL) and heated at 70 °C for 1 d to give a 0.64 mM solution of cage $[Pd_2L^5_4]^{4+}$.

^{1}H NMR (600 MHz, 298 K, CD_3CN): *δ* (ppm) = 10.09 (d, *J* = 2.4 Hz, 4H), 9.92 (dd, *J* = 5.4, 1.7 Hz, 4H), 9.78 (dd, *J* = 4.5, 1.8 Hz, 4H), 9.40 (d, *J* = 2.5 Hz, 4H), 8.56 (d, *J* = 2.5 Hz, 4H), 8.51 (dd, *J* = 8.3, 1.7 Hz, 4H), 8.49 (dd, *J* = 8.3, 1.8 Hz, 4H), 8.47 (d, *J* = 2.4 Hz, 4H), 8.13 (s, 8H), 7.97 (s, 8H), 7.94 (dd, *J* = 8.1, 4.5 Hz, 4H), 7.55 (dd, *J* = 8.1, 5.4 Hz, 4H), 2.39 (s, 24H), 1.85 (s, 16H).

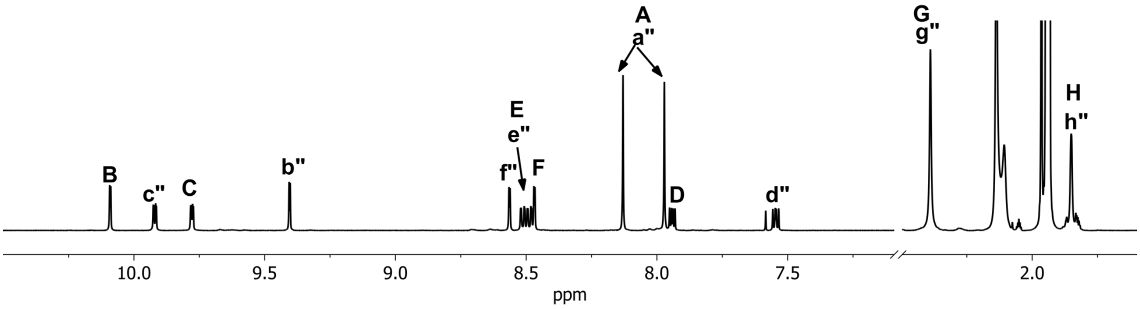

Figure S5 ^{1}H NMR spectrum (600 MHz, 298 K, CD_3CN) of $[Pd_2L^5_4]^{4+}$.

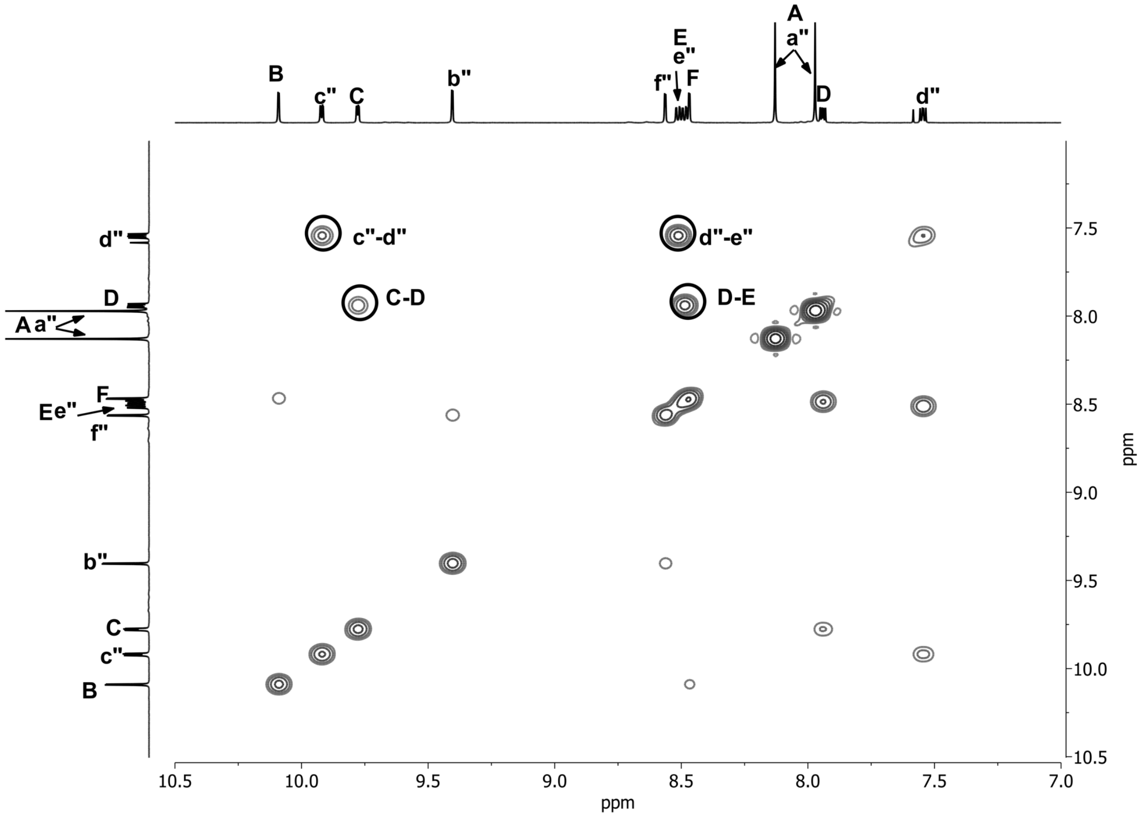

Figure S6 Partial ^{1}H – ^{1}H COSY spectrum (600 MHz, 298 K, CD_3CN) of $[Pd_2\mathbf{L}^{5}_{4}]^{4+}$.

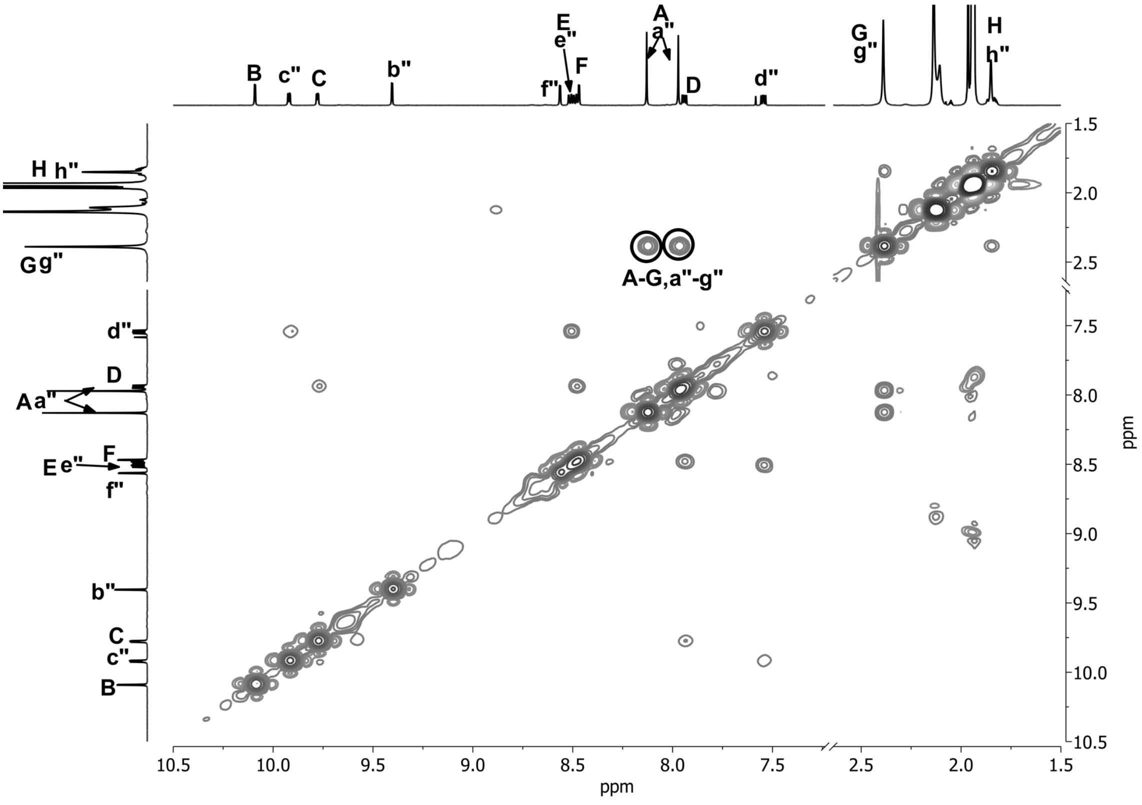

Figure S7 Partial ^{1}H – ^{1}H NOESY spectrum (600 MHz, 298 K, CD_3CN) of $[Pd_2\mathbf{L}^{5}_{4}]^{4+}$.

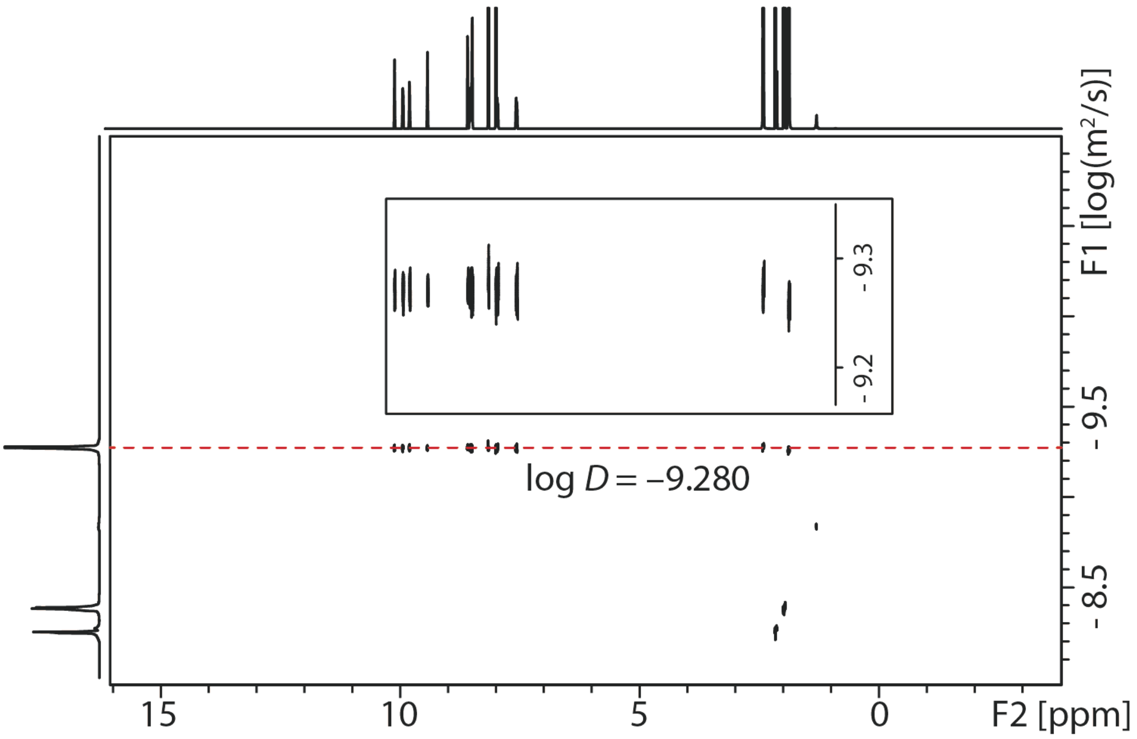

Figure S8 DOSY spectrum (500 MHz, 298 K, CD_3CN) of $[Pd_2\mathbf{L^5}_4]^{4+}$: diffusion coefficient = 5.3 x 10^{-10} m^2s^{-1}, log *D* = −9.28, r = 12.1 Å.

ESI HRMS ($C_{152}H_{96}N_{24}O_{16}Pd_2B_4F_{16}$): $[Pd_2\mathbf{L^5}_4]^{4+}$ calcd. for $C_{152}H_{96}N_{24}O_{16}Pd_2$ 681.6386; found 681.6375; $[Pd_2\mathbf{L^5}_4+BF_4]^{3+}$ calcd. for $C_{152}H_{96}N_{24}O_{16}Pd_2BF_4$ 937.8529; found 937.8503; $[Pd_2\mathbf{L^5}_4+2BF_4]^{2+}$ calcd. for $C_{152}H_{96}N_{24}O_{16}Pd_2B_2F_8$ 1450.2815; found 1450.2760.

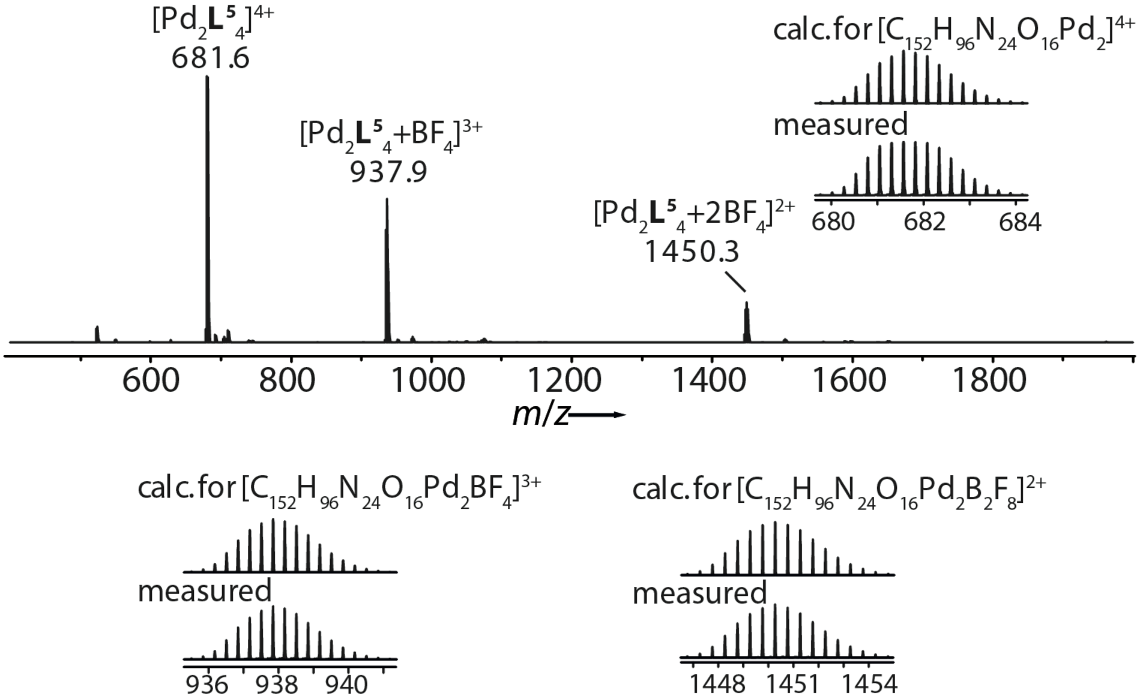

Figure S9 ESI mass spectrum of $[Pd_2\mathbf{L^5}_4]^{4+}$.

3.3 Formation and characterization of heteroleptic cage $[C_{70}@Pd_2\mathbf{L^2}_3\mathbf{L^5}]^{4+}$

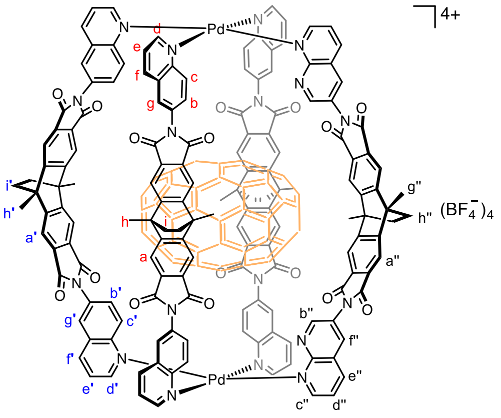

$[C_{70}@Pd_2\mathbf{L^2}_3\mathbf{L^5}]^{4+}$

A CD_3CN solution of bowl $[C_{70}@Pd_2\mathbf{L^2}_3(MeCN)_2](BF_4)_4$ (1000 µL, 0.64 mM, 0.64 µmol, 1 eq.) was combined with ligand $\mathbf{L^5}$ (0.4 mg, 0.64 µmol, 1 eq.) and heated at 70 °C for 1 d to give a 0.64 mM solution of heteroleptic cage $[C_{70}@Pd_2\mathbf{L^2}_3\mathbf{L^5}]^{4+}$.

^{1}H NMR (500 MHz, 298 K, CD_3CN): *δ* (ppm) = 9.74 – 9.67 (m, 8H), 9.54 (d, *J* = 9.3 Hz, 4H), 8.96 (d, *J* = 9.3 Hz, 2H), 8.71 – 8.64 (m, 2H), 8.59 – 8.54 (m, 6H), 8.50 (d, *J* = 2.5 Hz, 2H), 8.35 (d, *J* = 2.5 Hz, 2H), 8.09 (s, 4H), 8.06 (s, 4H), 8.04 – 8.01 (m, 6H), 7.89 (s, 4H), 7.87 – 7.81 (m, 6H), 7.69 (dd, *J* = 8.3, 5.4 Hz, 6H), 7.28 (dd, *J* = 9.3, 2.3 Hz, 4H), 7.11 (dd, *J* = 9.3, 2.3 Hz, 2H), 2.39 (s, 6H), 2.35 (s, 6H), 2.26 (s, 12H), 1.87 (s, 8H), 1.80 (m, 8H).

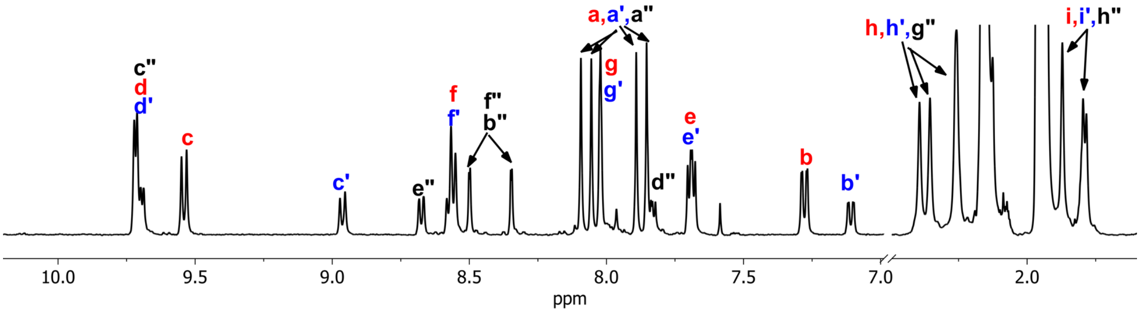

Figure S10 ^{1}H NMR spectrum (500 MHz, 298 K, CD_3CN) of $[C_{70}@Pd_2\mathbf{L^2}_3\mathbf{L^5}]^{4+}$.

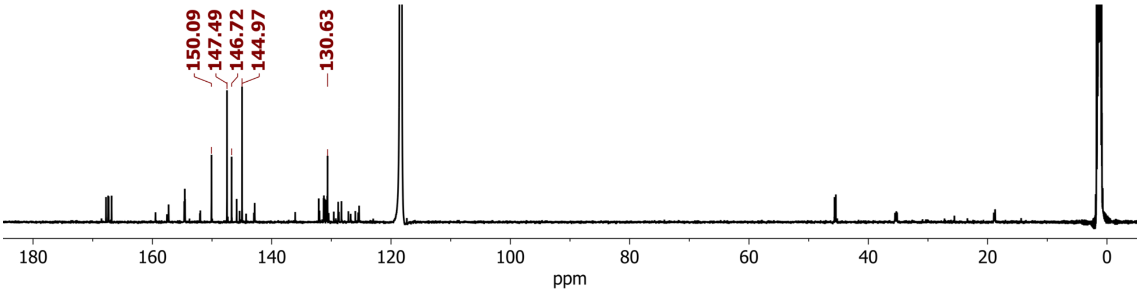

Figure S11 ^{13}C NMR spectrum (151 MHz, 298 K, CD_3CN) of $[C_{70}@Pd_2\mathbf{L^2}_3\mathbf{L^5}]^{4+}$. Five single signals at 150.09, 147.49, 146.72, 144.97, 130.63 ppm correspond to the encapsulated C_{70}.

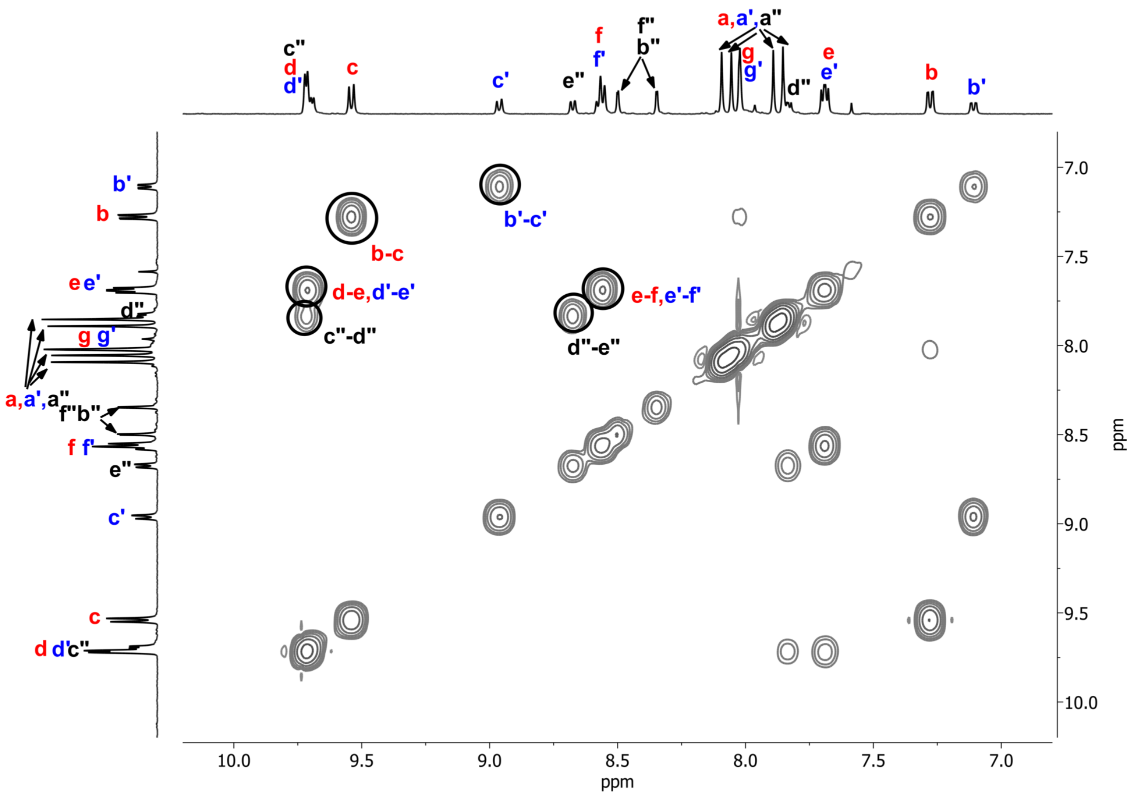

Figure S12 Partial ^{1}H – ^{1}H COSY spectrum (500 MHz, 298 K, CD_3CN) of $[C_{70}@Pd_2\mathbf{L^2}_3\mathbf{L^5}]^{4+}$.

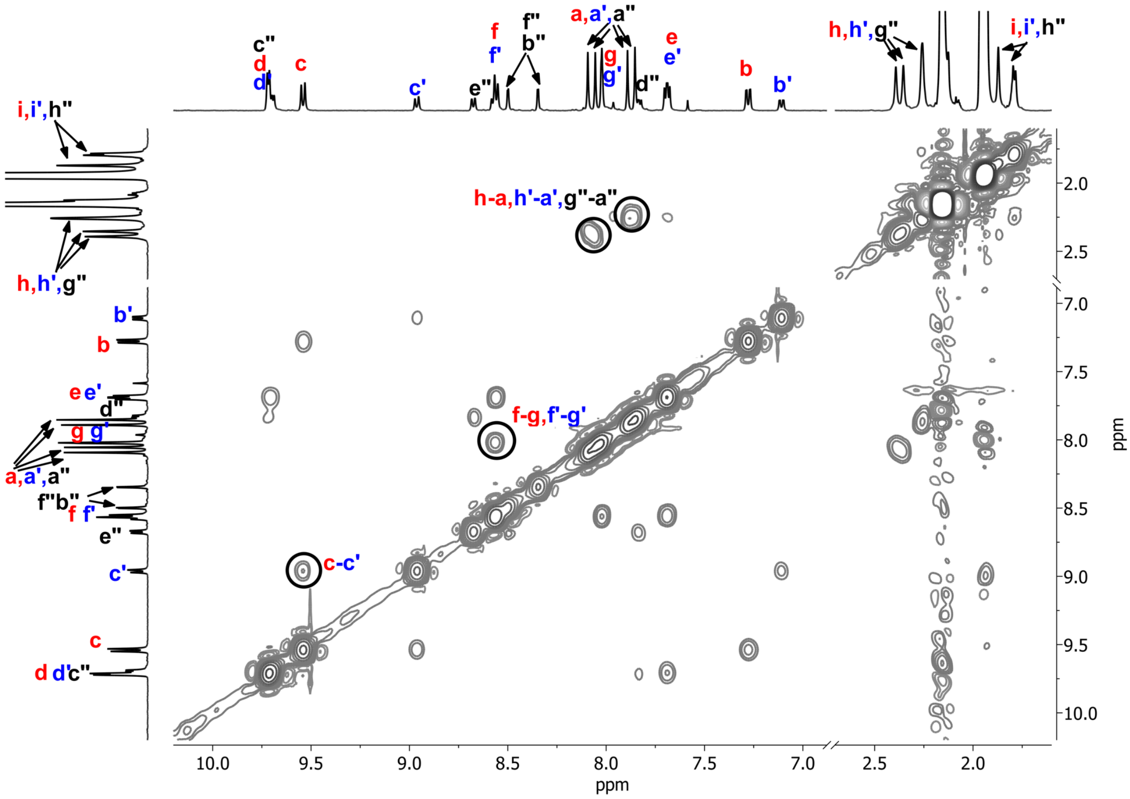

Figure S13 Partial ^{1}H – ^{1}H NOESY spectrum (500 MHz, 298 K, CD_3CN) of $[C_{70}@Pd_2\mathbf{L^2}_3\mathbf{L^5}]^{4+}$.

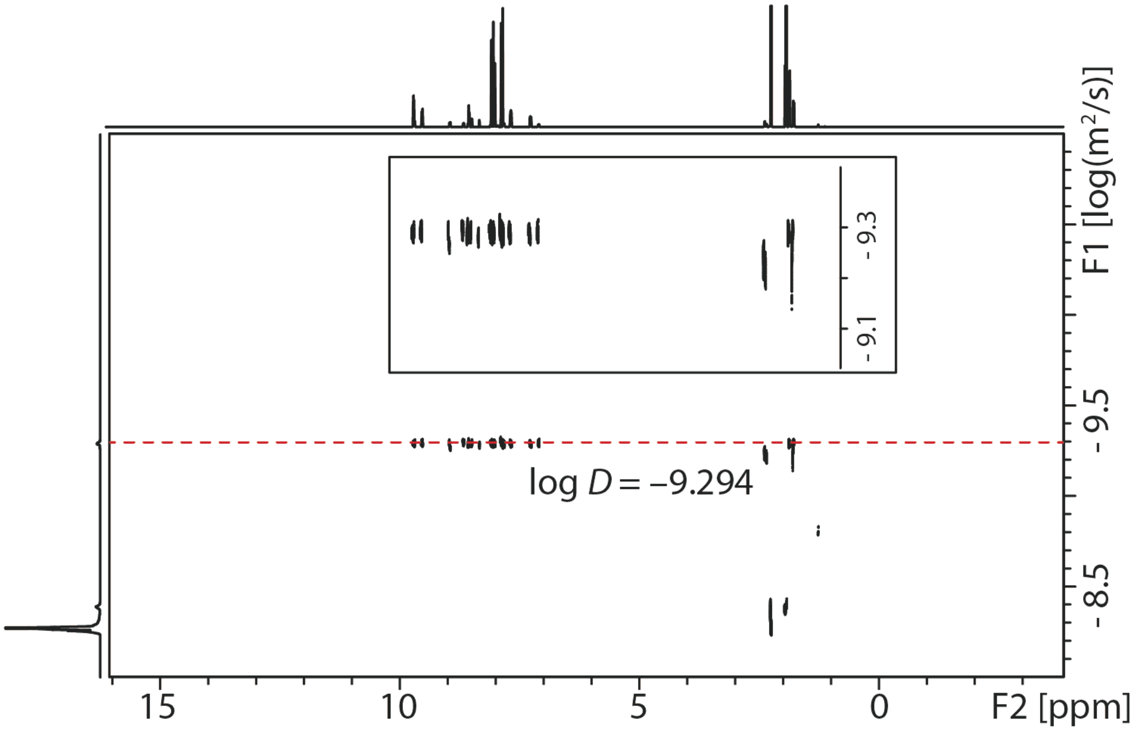

Figure S14 DOSY spectrum (500 MHz, 298 K, CD_3CN) of $[C_{70}@Pd_2\mathbf{L^2}_3\mathbf{L^5}]^{4+}$: diffusion coefficient = 5.1 x 10^{-10} m^2s^{-1}, log *D* = −9.29, r = 12.5 Å.

ESI HRMS ($C_{228}H_{102}N_{18}O_{16}Pd_2B_4F_{16}$): $[C_{70}@Pd_2\mathbf{L^2}_3\mathbf{L^5}]^{4+}$ calcd. for $C_{228}H_{102}N_{18}O_{16}Pd_2$ 890.6467; found 890.6458; $[C_{70}@Pd_2\mathbf{L^2}_3\mathbf{L^5}+BF_4]^{3+}$ calcd. for $C_{228}H_{102}N_{18}O_{16}Pd_2BF_4$ 1216.1967; found 1216.1948; $[C_{70}@Pd_2\mathbf{L^2}_3\mathbf{L^5}+2BF_4]^{2+}$ calcd. for $C_{228}H_{102}N_{18}O_{16}Pd_2B_2F_8$ 1867.7973; found 1867.7915.

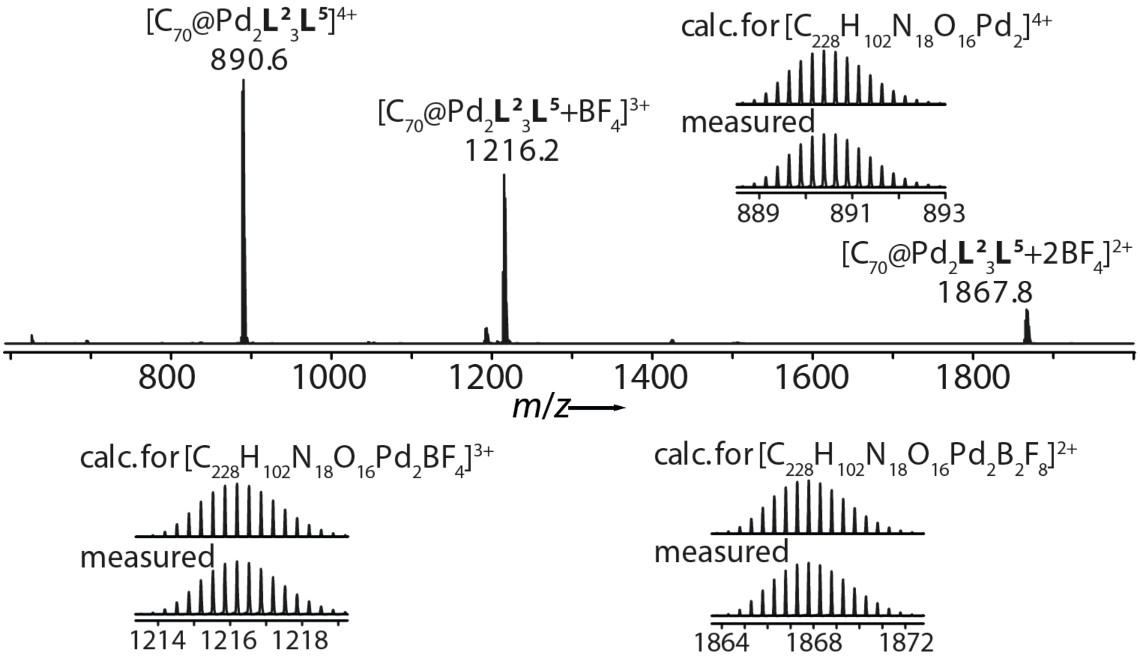

Figure S15 ESI mass spectrum of $[C_{70}@Pd_2\mathbf{L^2}_3\mathbf{L^5}]^{4+}$.

3.4 Formation and characterization of heteroleptic cage [C_{60}@Pd_2**L**$^2_2$**L**$^5_2$]$^{4+}$

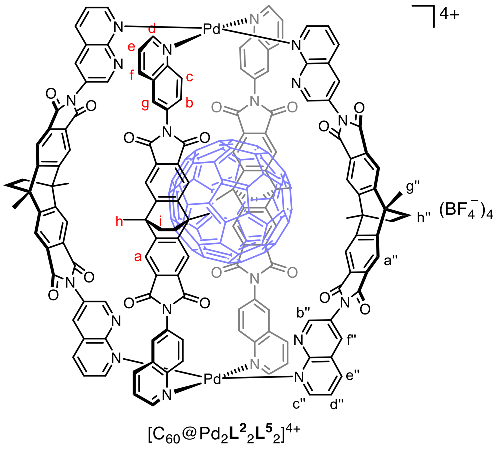

A solution of ligand **L**^{2} (1823 µL, 5 mM/CH_2Cl_2, 9.11 µmol, 1 eq.) was combined with another solution of ligand **L**^{5} (1823 µL, 5 mM/CH_2Cl_2, 9.11 µmol, 1 eq.), followed by removing CH_2Cl_2 from the vessel in vacuum. Then a solution of [Pd(MeCN)$_4$](BF$_4$)$_2$ (607.6 µL, 15 mM/CD_3CN, 9.11 µmol, 1 eq.), excess C_{60} (6.6 mg, 9.2 µmol, 1 eq.) and CD_3CN (6510 µL) were added into the vessel and stirred at 70 °C for 2 d. Excess C_{60} solid was removed by filtration to give a 0.64 mM pale purple solution of heteroleptic cage [C_{60}@Pd_2**L**$^2_2$**L**$^5_2$]$^{4+}$.

^{1}H NMR (500 MHz, 298 K, CD_3CN): δ (ppm) = 11.00 (d, J = 9.3 Hz, 4H), 9.82 (dd, J = 5.3, 1.7 Hz, 4H), 9.75 (d, J = 5.4 Hz, 4H), 8.68 – 8.62 (m, 8H), 8.52 – 8.47 (m, 8H), 7.92 (m, 20H), 7.83 (dd, J = 8.2, 5.4 Hz, 4H), 7.69 (dd, J = 8.3, 5.4 Hz, 4H), 7.35 (dd, J = 9.3, 2.2 Hz, 4H), 2.26 (m, 24H), 1.79 (m, 16H).

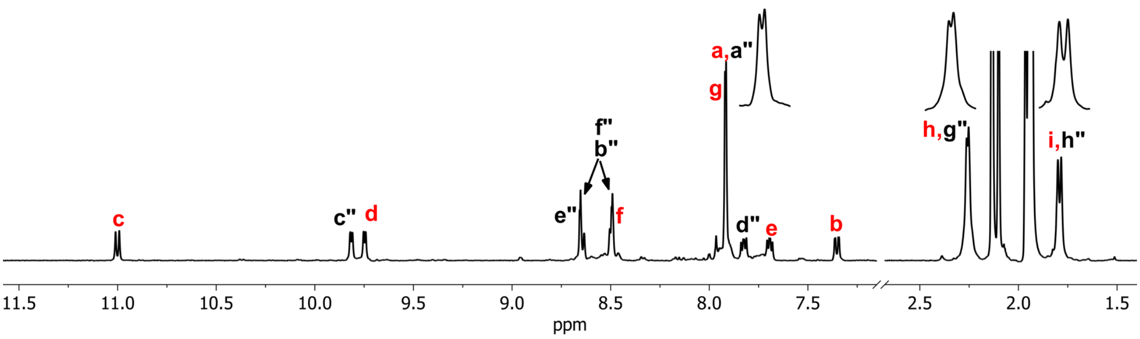

Figure S16 ^{1}H NMR spectrum (500 MHz, 298 K, CD_3CN) of [C_{60}@Pd_2**L**$^2_2$**L**$^5_2$]$^{4+}$.

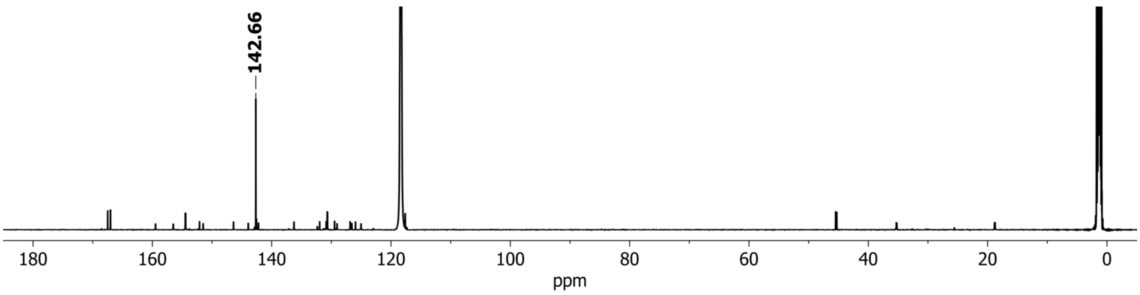

Figure S17 ^{13}C NMR spectrum (151 MHz, 298 K, CD_3CN) of [C_{60}@Pd_2**L**$^2_2$**L**$^5_2$]$^{4+}$. A single signal at 142.66 ppm corresponds to the encapsulated C_{60}.

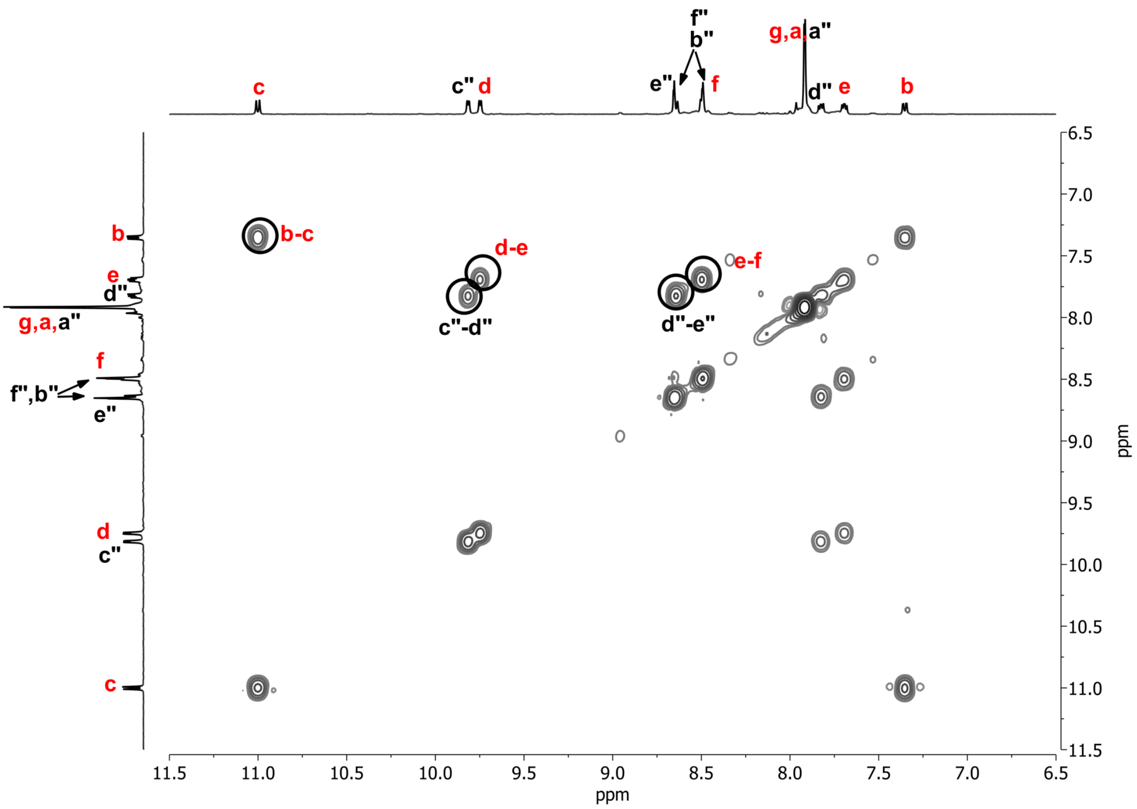

Figure S18 Partial ^{1}H – ^{1}H COSY spectrum (500 MHz, 298 K, CD_3CN) of $[C_{60}@Pd_2\mathbf{L^2}_2\mathbf{L^5}_2]^{4+}$.

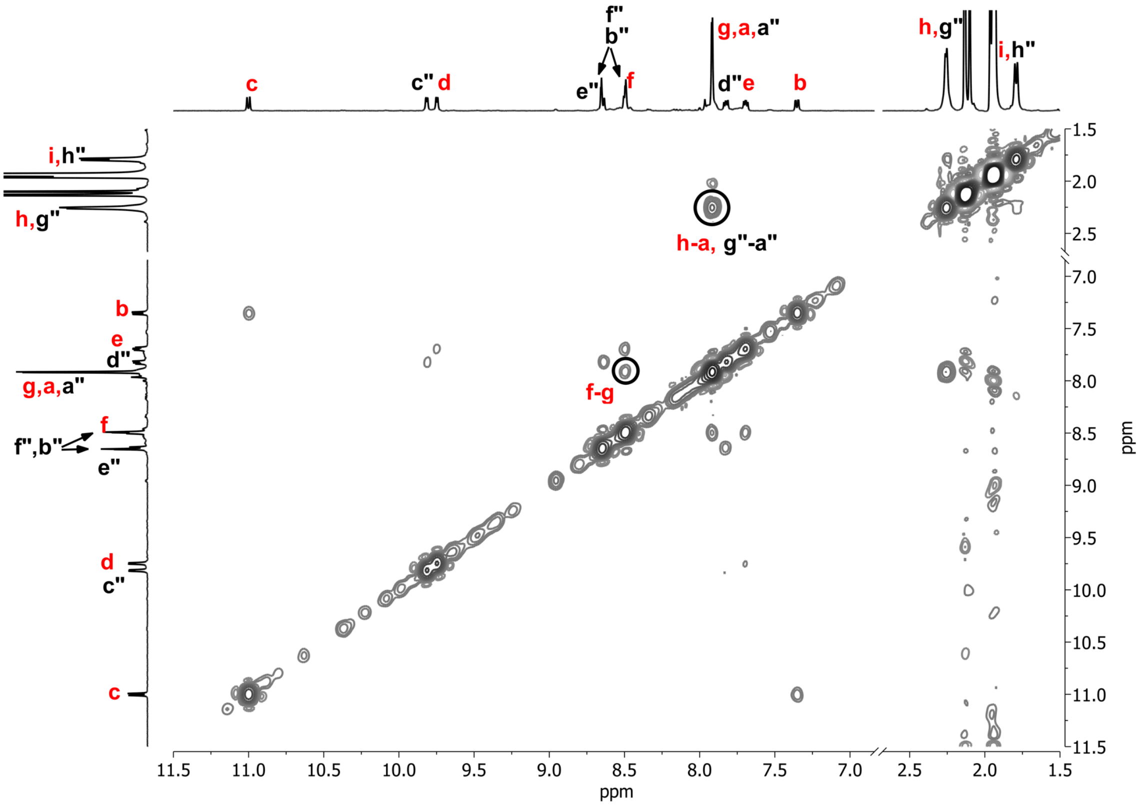

Figure S19 Partial ^{1}H – ^{1}H NOESY spectrum (500 MHz, 298 K, CD_3CN) of $[C_{60}@Pd_2\mathbf{L^2}_2\mathbf{L^5}_2]^{4+}$.

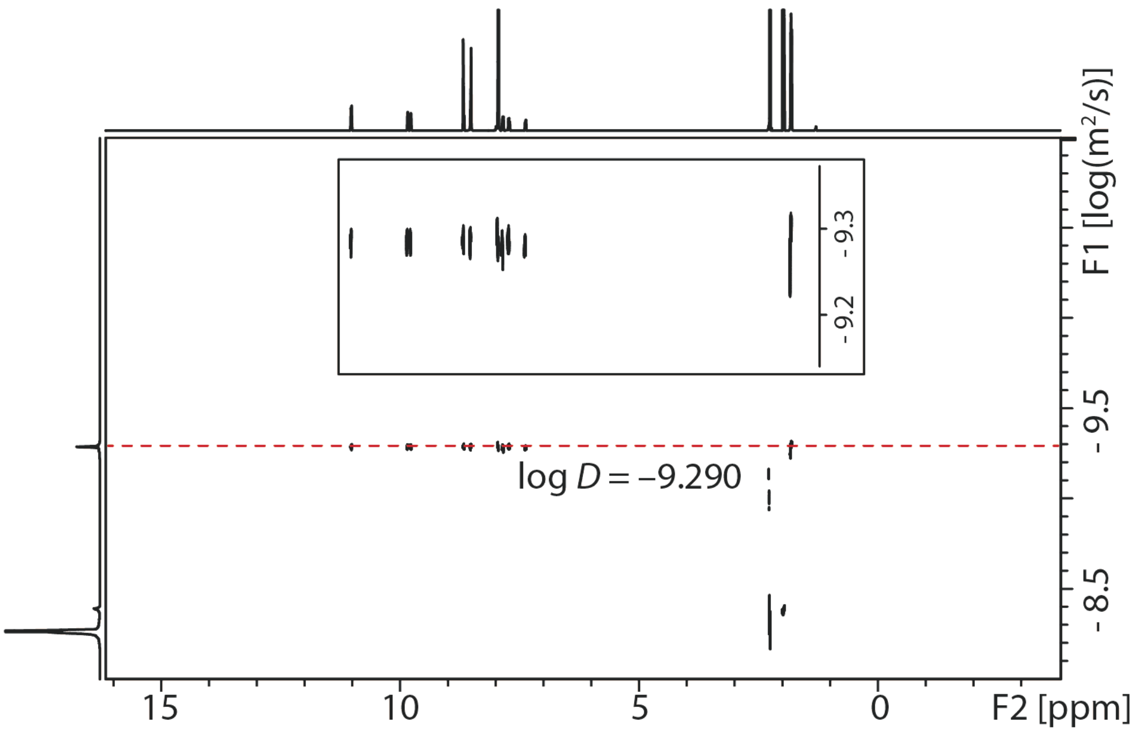

Figure S20 DOSY spectrum (500 MHz, 298 K, CD_3CN) of $[C_{60}@Pd_2\mathbf{L^2}_2\mathbf{L^5}_2]^{4+}$: diffusion coefficient = 5.1 x 10^{-10} m^2s^{-1}, log D = −9.29, r = 12.4 Å.

ESI HRMS ($C_{216}H_{100}N_{20}O_{16}Pd_2B_4F_{16}$): $[C_{60}@Pd_2\mathbf{L^2}_2\mathbf{L^5}_2]^{4+}$ calcd. for $C_{216}H_{100}N_{20}O_{16}Pd_2$ 860.8940; found 860.8911; $[C_{60}@Pd_2\mathbf{L^2}_2\mathbf{L^5}_2+BF_4]^{3+}$ calcd. for $C_{216}H_{100}N_{20}O_{16}Pd_2BF_4$ 1176.8601; found 1176.8556; $[C_{60}@Pd_2\mathbf{L^2}_2\mathbf{L^5}_2+2BF_4]^{2+}$ calcd. for $C_{216}H_{100}N_{20}O_{16}Pd_2B_2F_8$ 1808.7923; found 1808.7824.

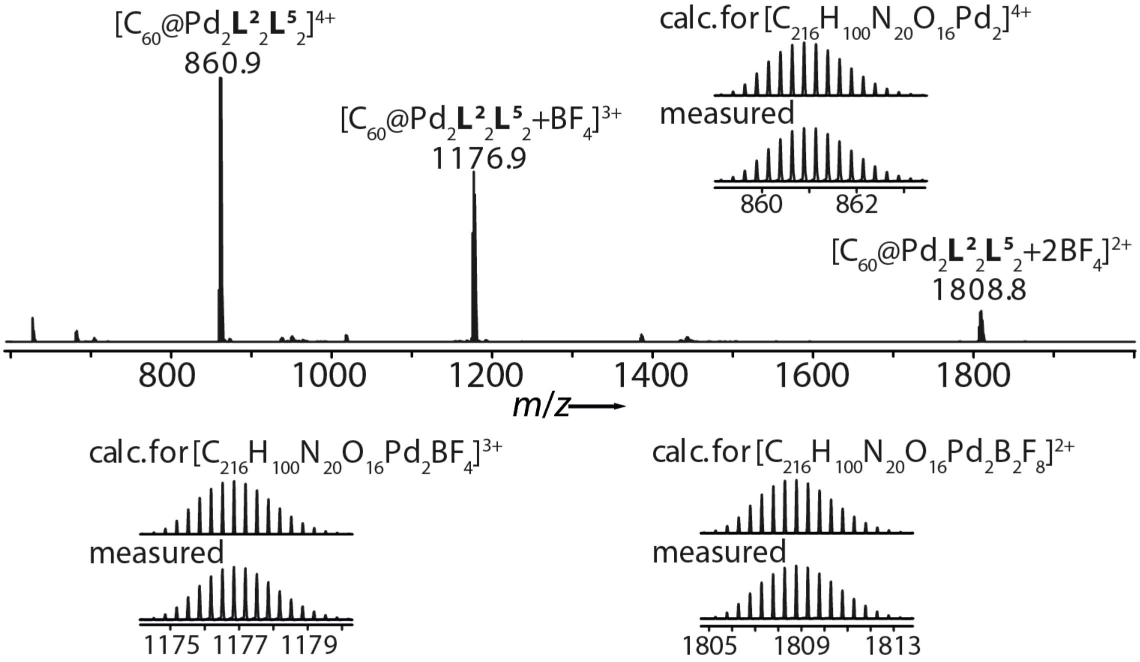

Figure S21 ESI mass spectrum of $[C_{60}@Pd_2\mathbf{L^2}_2\mathbf{L^5}_2]^{4+}$.

Table S1 Comparison of the chemical shift of quinoline proton (H_c) in different species (CD_3CN, 298K).

Species	Chemical shift (ppm)
$\mathbf{L^2}$	8.16
$[Pd_2\mathbf{L^2}_3(MeCN)_2]^{4+}$	9.99, 9.31
$[Pd_2\mathbf{L^2}_4]^{4+}$	9.54
$[C_{60}@Pd_2\mathbf{L^2}_3(MeCN)_2]^{4+}$	10.32, 9.07
$[C_{70}@Pd_2\mathbf{L^2}_3(MeCN)_2]^{4+}$	9.93, 8.74
$[C_{70}@Pd_2\mathbf{L^2}_4]^{4+}$	8.74
$[C_{70}@Pd_2\mathbf{L^2}_3\mathbf{L^5}]^{4+}$	9.54, 8.96
$[C_{60}@Pd_2\mathbf{L^2}_2\mathbf{L^5}_2]^{4+}$	11.00

3.5 Formation and characterization of heteroleptic cage $[C_{60}@Pd_2L^4_2L^5(MeCN)_2]^{4+}$

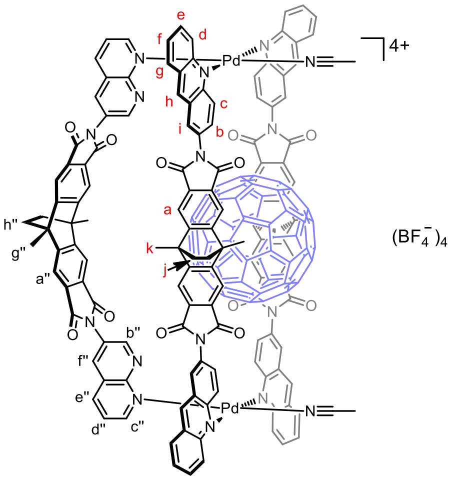

$[C_{60}@Pd_2\mathbf{L^4}_2\mathbf{L^5}(MeCN)_2]^{4+}$

A solution of ligand **L⁴** (911 µL, 5 mM/CH_2Cl_2, 4.56 µmol, 2 eq.) was combined with another solution of ligand **L⁵** (456 µL, 5 mM/CH_2Cl_2, 2.28 µmol, 1 eq.), followed by removing CH_2Cl_2 from the vessel in vacuum. Then a solution of $[Pd(MeCN)_4](BF_4)_2$ (304 µL, 15 mM/CD_3CN, 4.56 µmol, 2 eq.), excess C_{60} (4.5 mg, 6.3 µmol, 1.4 eq.) and CD_3CN (3255 µL) were added into the vessel and stirred at 70 °C for 1 d. Excess C_{60} solid was removed by filtration to give a pale purple solution, wherein the heteroleptic bowl $[C_{60}@Pd_2\mathbf{L^4}_2\mathbf{L^5}(MeCN)_2]^{4+}$ exists as the major species.

^{1}H NMR (600 MHz, 298 K, CD_3CN): δ (ppm) = 10.93 (d, J = 9.3 Hz, 4H), 10.61 (d, J = 8.9 Hz, 4H), 9.51 (s, 4H), 9.46 (dd, J = 5.7, 1.6 Hz, 2H), 8.71 (ddd, J = 8.5, 6.8, 1.4 Hz, 4H), 8.44 (d, J = 2.6 Hz, 2H), 8.40 – 8.34 (m, 10H), 8.27 (d, J = 2.5 Hz, 2H), 8.08 (dd, J = 9.3, 2.3 Hz, 4H), 8.04 – 7.98 (m, 12H), 7.83 (s, 4H), 7.43 (dd, J = 8.1, 5.8 Hz, 2H), 2.28 (s, 13H), 2.24 (d, J = 2.3 Hz, 5H), 2.20 (s, 4H), 1.85 – 1.81 (m, 13H), 1.73 (d, J = 5.8 Hz, 4H). The proton signals (H_j, H_k, $H_{g''}$, $H_{h''}$) in the aliphatic region are heavily overlapped and it is difficult to list the exact chemical shifts and integration of these signals. All the major signals in the aromatic region could be assigned via 2D NMR spectroscopy.

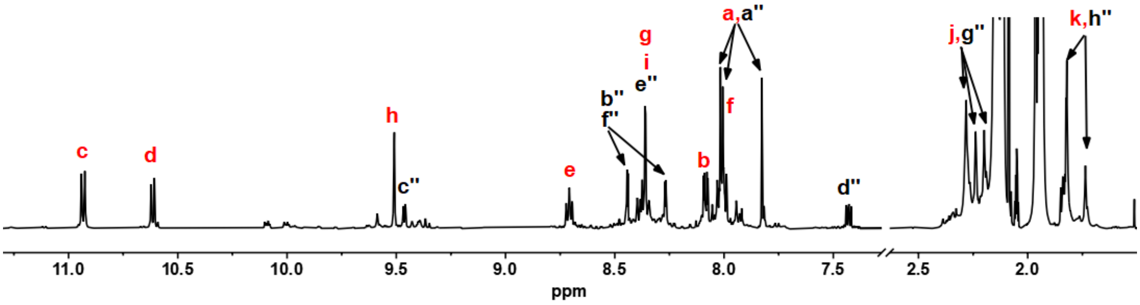

Figure S22 ^{1}H NMR spectrum (600 MHz, 298 K, CD_3CN) of $[C_{60}@Pd_2\mathbf{L^4}_2\mathbf{L^5}(MeCN)_2]^{4+}$.

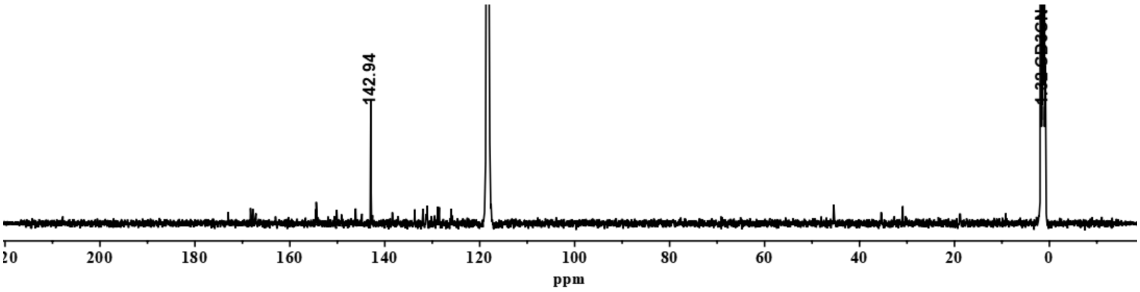

Figure S23 ^{13}C NMR spectrum (151 MHz, 298 K, CD_3CN) of $[C_{60}@Pd_2\mathbf{L^4}_2\mathbf{L^5}(MeCN)_2]^{4+}$. A single signal at 142.94 ppm corresponds to the encapsulated C_{60}.

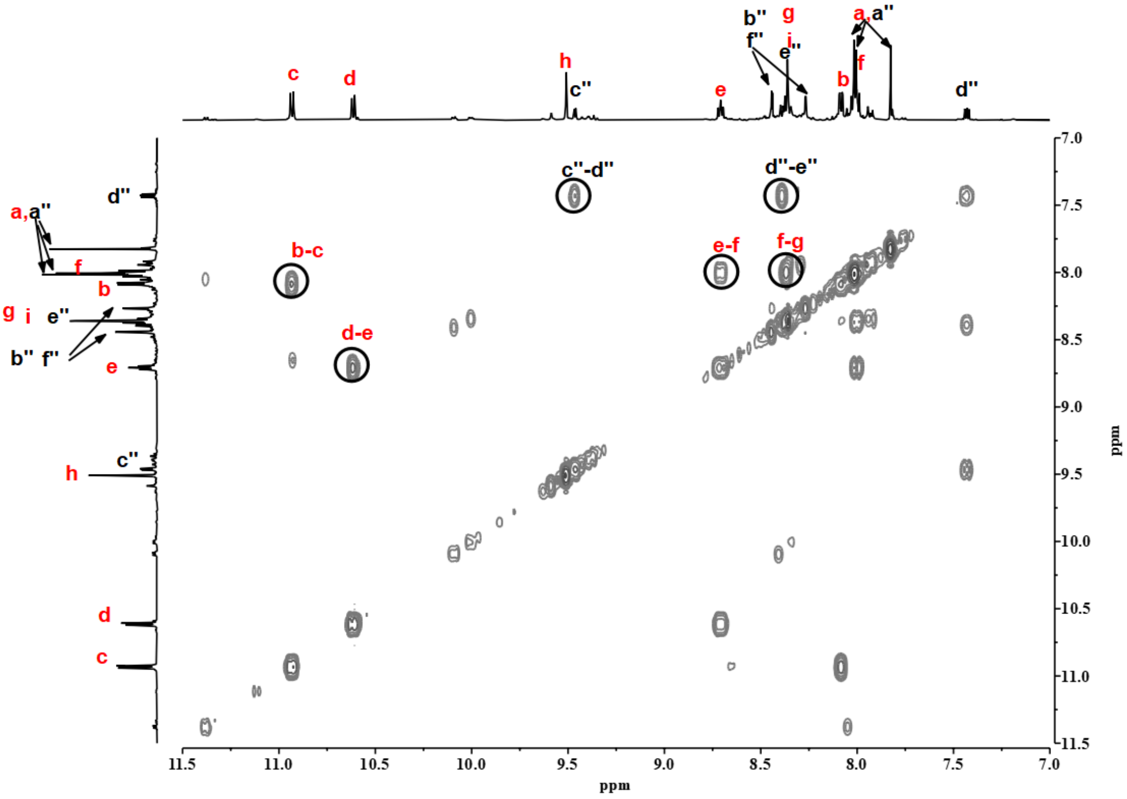

Figure S24 Partial ^{1}H – ^{1}H COSY spectrum (600 MHz, 298 K, CD_3CN) of $[C_{60}@Pd_2\mathbf{L^4}_2\mathbf{L^5}(MeCN)_2]^{4+}$.

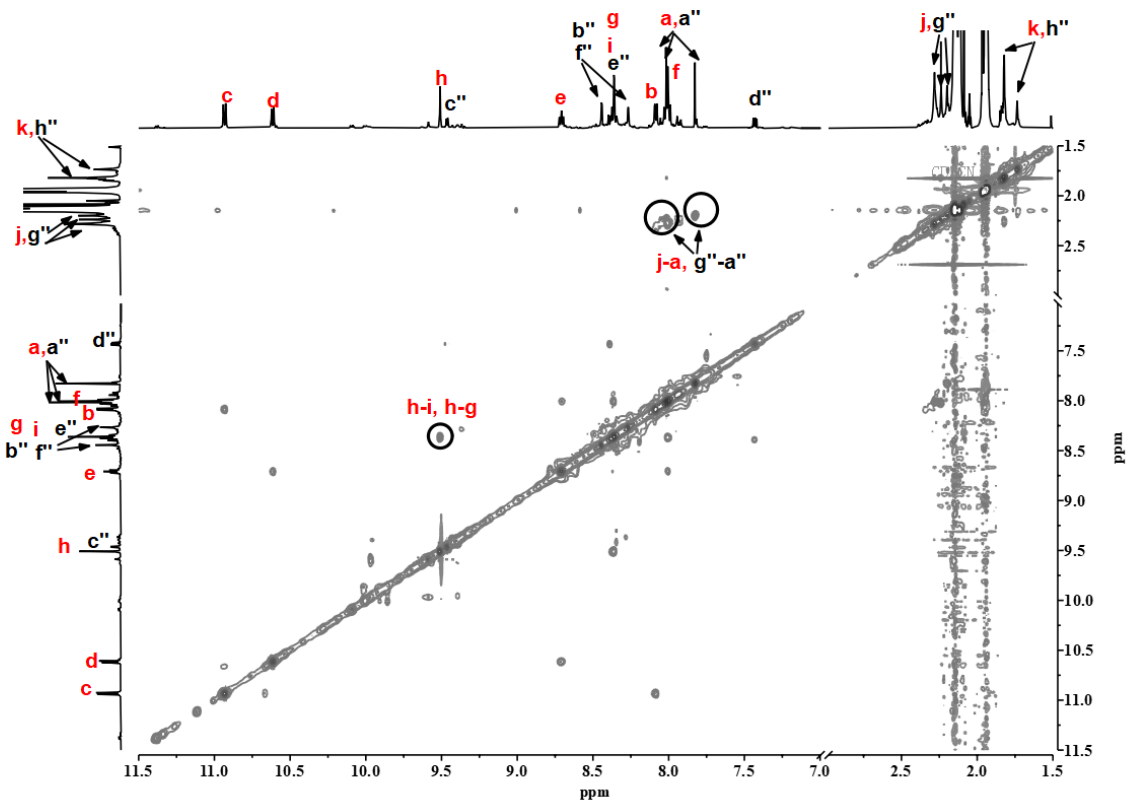

Figure S25 Partial $^{1}H - ^{1}H$ NOESY spectrum (600 MHz, 298 K, CD_3CN) of $[C_{60}@Pd_2\mathbf{L^4}_2\mathbf{L^5}(MeCN)_2]^{4+}$.

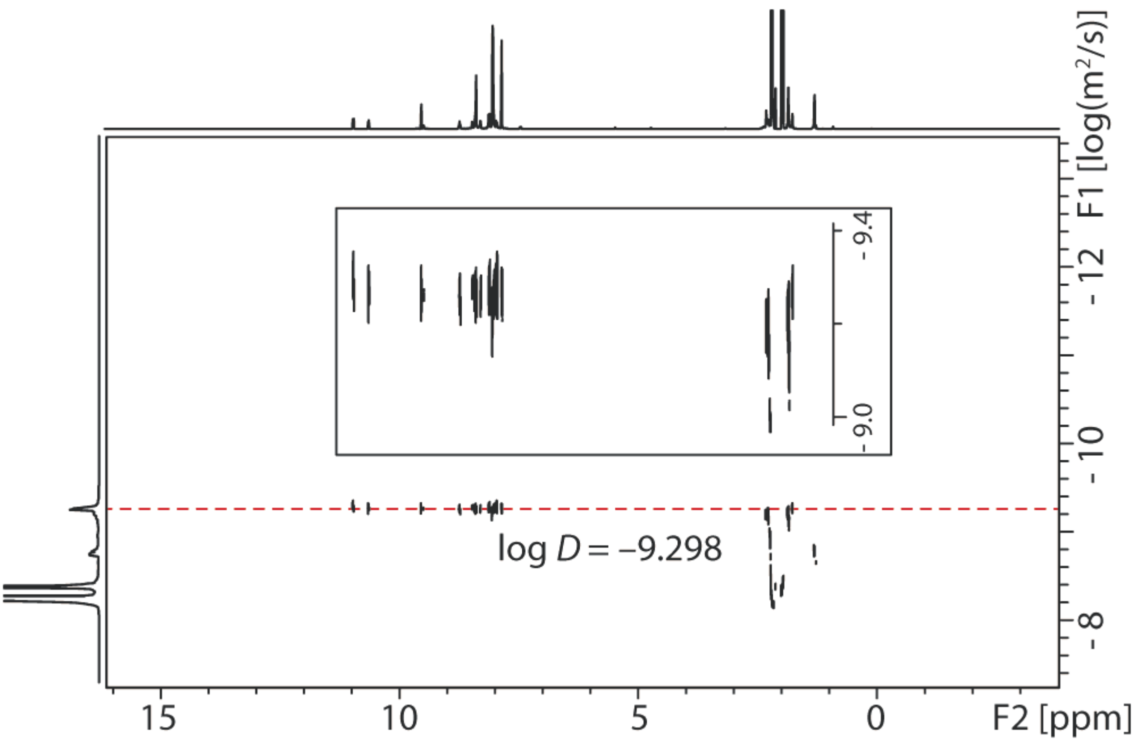

Figure S26 DOSY spectrum (500 MHz, 298 K, CD_3CN) of $[C_{60}@Pd_2\mathbf{L^4}_2\mathbf{L^5}(MeCN)_2]^{4+}$: diffusion coefficient = 5.0×10^{-10} m^2s^{-1}, log D = −9.30, r = 12.6 Å.

ESI HRMS ($C_{198}H_{90}N_{16}O_{12}Pd_2B_4F_{16}$): [$C_{60}$@$Pd_2$**$L^4$**$_2$**$L^5$**$(MeCN)_2$]$^{4+}$ calcd. for $C_{198}H_{90}N_{16}O_{12}Pd_2$ 774.3764; found 774.3775; [C_{60}@Pd_2**L^4**$_2$**L^5**(MeCN)+F]$^{3+}$ calcd. for $C_{196}H_{87}N_{15}O_{12}Pd_2F$ 1025.1593; found 1025.1605; [C_{60}@Pd_2**L^4**$_2$**L^5**(MeCN)+Cl]$^{3+}$ calcd. for $C_{196}H_{87}N_{15}O_{12}Pd_2Cl$ 1030.4825; found 1030.4840; [C_{60}@Pd_2**L^4**$_2$**L^5**$(MeCN)_2$+BF_4]$^{3+}$ calcd. for $C_{198}H_{90}N_{16}O_{12}Pd_2BF_4$ 1061.5032; found 1061.5046.

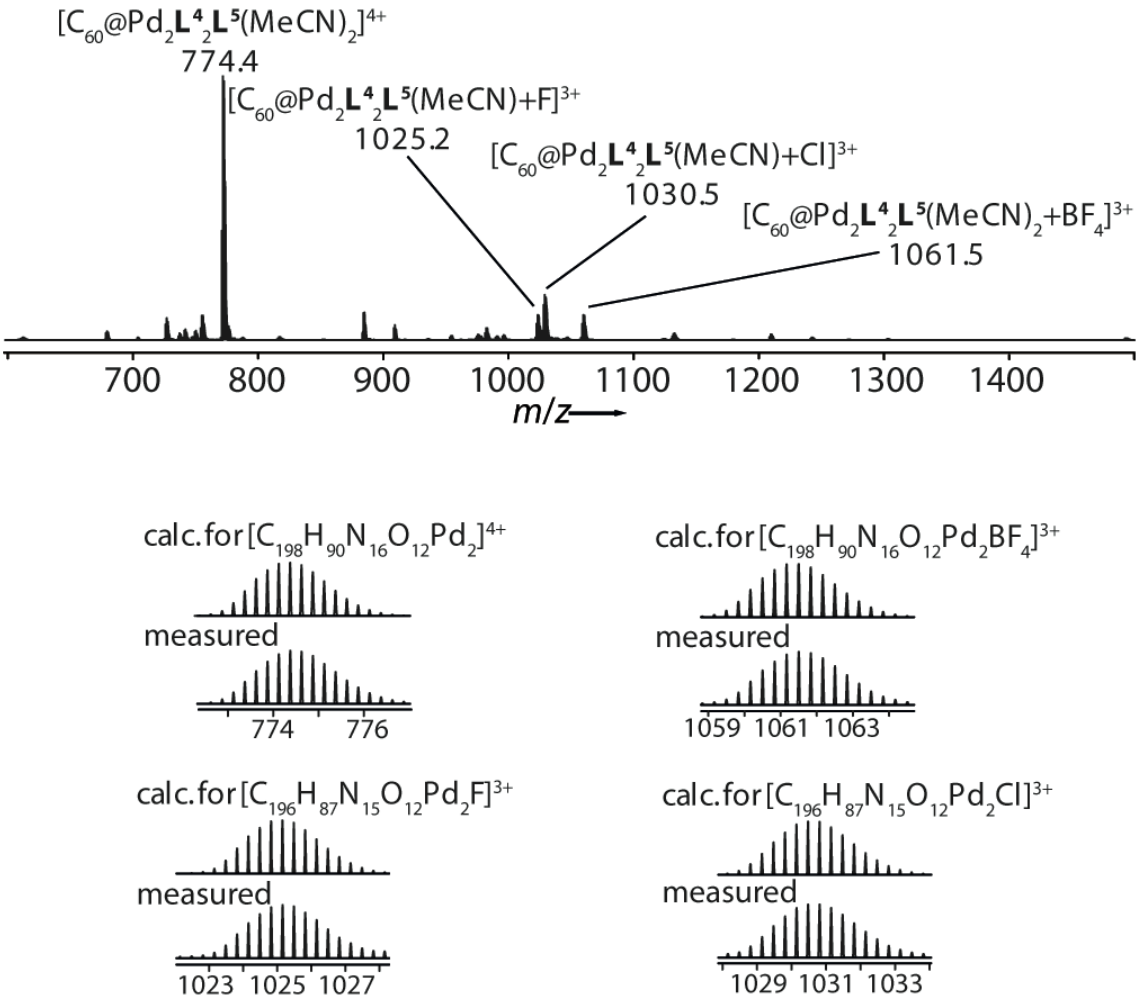

Figure S27 ESI mass spectrum of [C_{60}@Pd_2**L^4**$_2$**L^5**$(MeCN)_2$]$^{4+}$.

3.6 Formation and characterization of heteroleptic cage $[C_{70}@Pd_2\mathbf{L^4}_2\mathbf{L^5}(MeCN)_2]^{4+}$

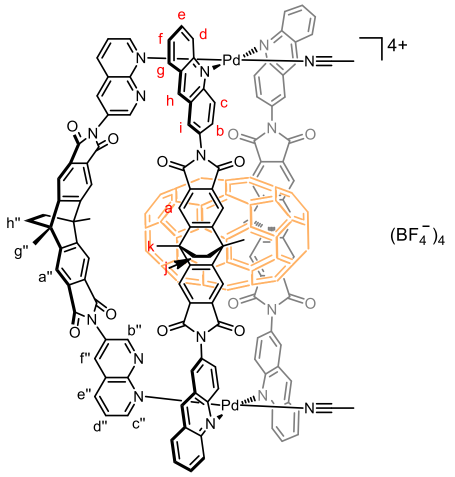

$[C_{70}@Pd_2\mathbf{L^4}_2\mathbf{L^5}(MeCN)_2]^{4+}$

A solution of ligand $\mathbf{L^4}$ (2812 µL, 1.67 mM/CH_2Cl_2, 4.70 µmol, 2 eq.) was combined with another solution of ligand $\mathbf{L^5}$ (1406 µL, 1.67 mM/CH_2Cl_2, 2.35 µmol, 1 eq.), followed by removing CH_2Cl_2 from the vessel in vacuum. Then a solution of $[Pd(MeCN)_4](BF_4)_2$ (312 µL, 15 mM/CD_3CN, 4.68 µmol, 2 eq.), excess C_{70} (3.8 mg, 4.5 µmol, 1.8 eq.) and CD_3CN (3348 µL) were added into the vessel and stirred at 70 °C for 1 d. Excess C_{70} solid was removed by filtration to give a brown solution, wherein the heteroleptic bowl $[C_{70}@Pd_2\mathbf{L^4}_2\mathbf{L^5}(MeCN)_2]^{4+}$ exists as the major species.

Most proton signals of the heteroleptic bowl $[C_{70}@Pd_2\mathbf{L^4}_2\mathbf{L^5}(MeCN)_2]^{4+}$ are heavily overlapping and it is difficult to list the exact chemical shifts and integration of these signals. A majority of the signals in the aromatic region could be assigned via 2D NMR spectroscopy.

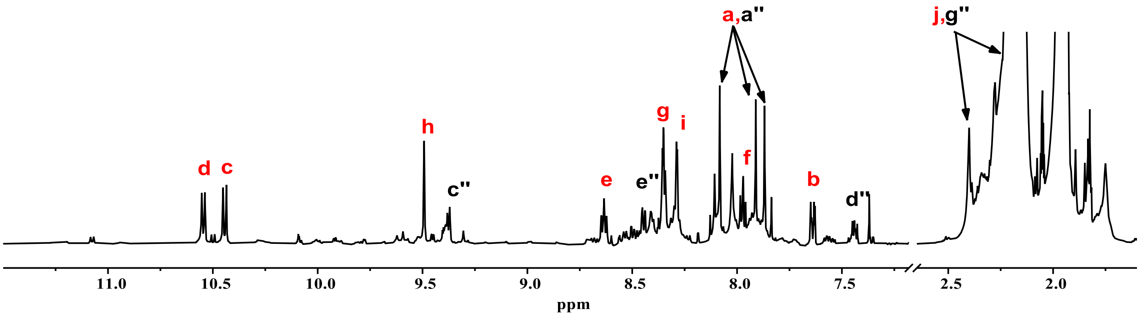

Figure S28 ^1H NMR spectrum (600 MHz, 298 K, CD_3CN) of $[C_{70}@Pd_2\mathbf{L^4}_2\mathbf{L^5}(MeCN)_2]^{4+}$.

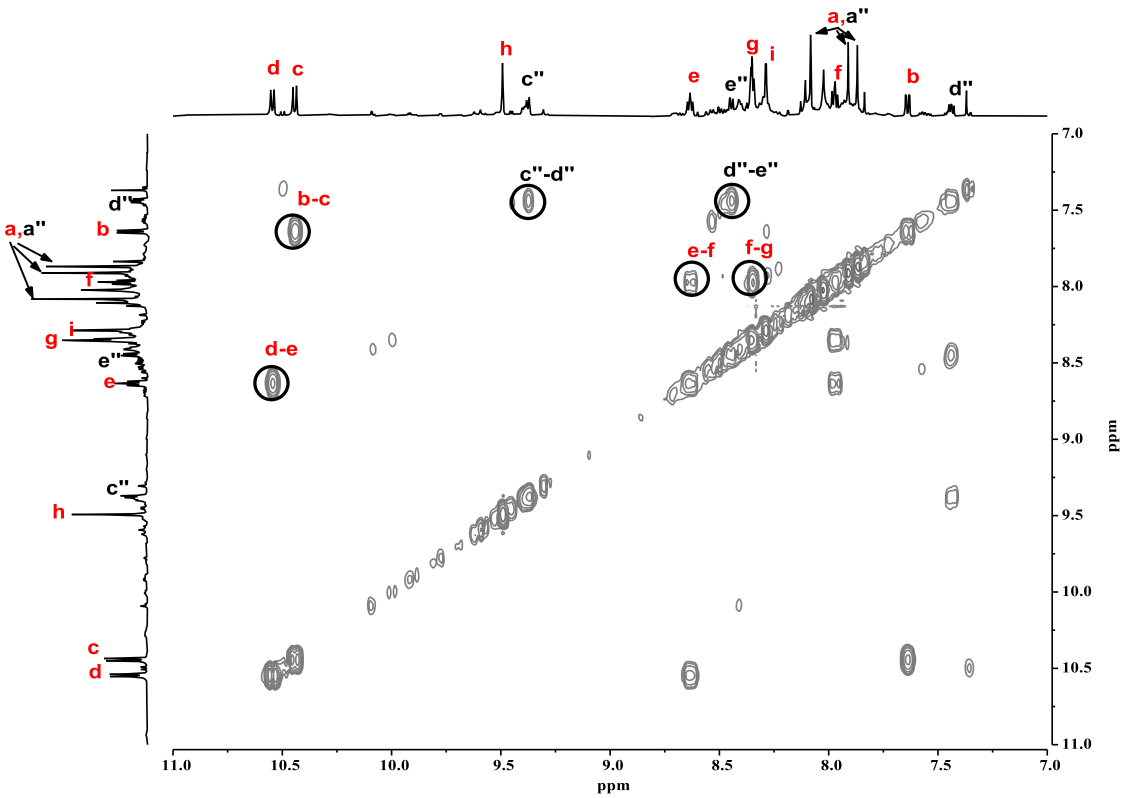

Figure S29 Partial $^1H - ^1H$ COSY spectrum (600 MHz, 298 K, CD_3CN) of $[C_{70}@Pd_2\mathbf{L^4}_2\mathbf{L^5}(MeCN)_2]^{4+}$.

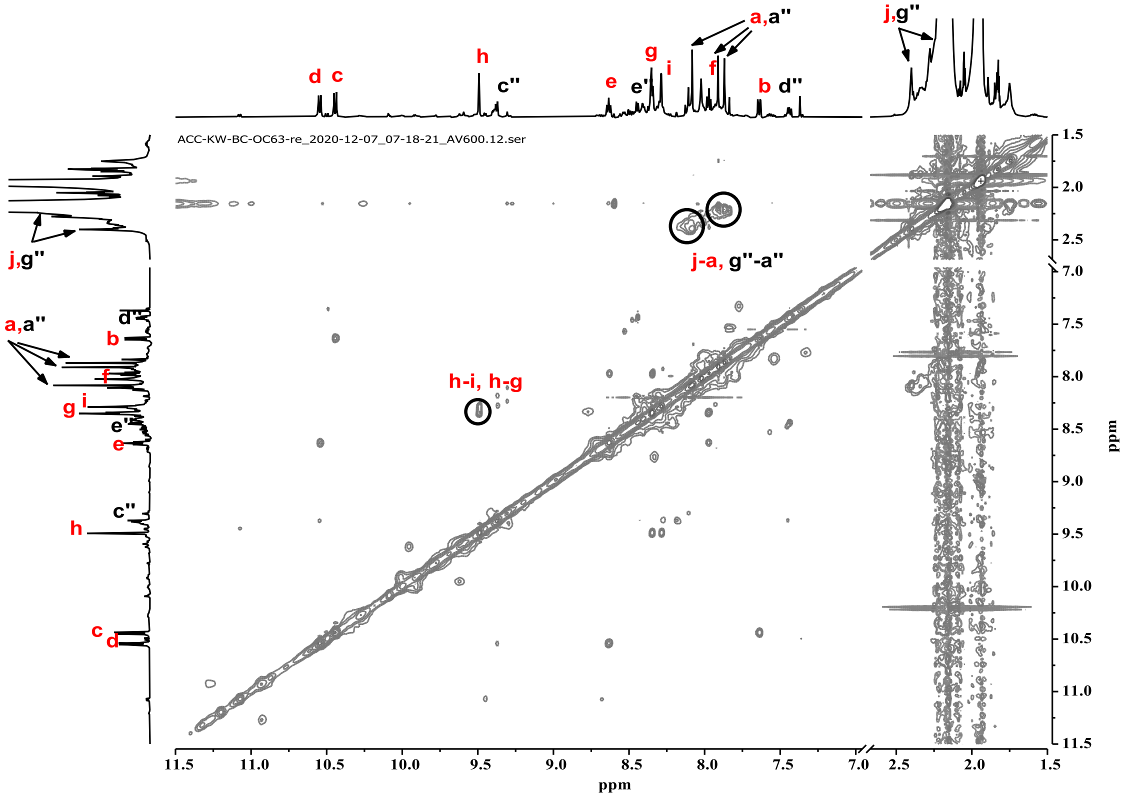

Figure S30 Partial $^1H - ^1H$ NOESY spectrum (600 MHz, 298 K, CD_3CN) of $[C_{70}@Pd_2\mathbf{L^4}_2\mathbf{L^5}(MeCN)_2]^{4+}$.

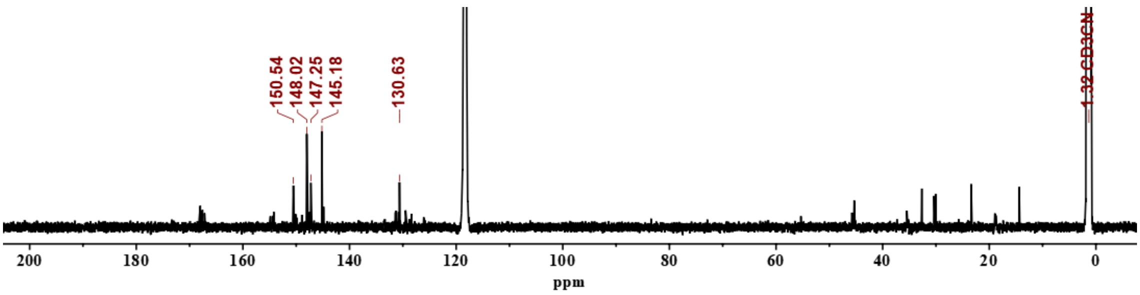

Figure S31 ^{13}C NMR spectrum (151 MHz, 298 K, CD_3CN) of $[C_{70}@Pd_2\mathbf{L^4}_2\mathbf{L^5}(MeCN)_2]^{4+}$. Five single signals at 150.54, 148.02, 147.25, 145.18, 130.63 ppm correspond to the encapsulated C_{70}.

ESI HRMS ($C_{208}H_{90}N_{16}O_{12}Pd_2B_4F_{16}$): $[Pd_2\mathbf{L^4}_2\mathbf{L^5}]^{4+}$ calcd. for $C_{134}H_{84}N_{14}O_{12}Pd_2$ 549.1021; found 549.0970; $[Pd_2\mathbf{L^4}_2\mathbf{L^5}(MeCN)]^{4+}$ calcd. for $C_{136}H_{87}N_{15}O_{12}Pd_2$ 559.3588; found 559.3537; $[Pd_2\mathbf{L^4}_2\mathbf{L^5}(MeCN)_2]^{4+}$ calcd. for $C_{138}H_{90}N_{16}O_{12}Pd_2$ 569.6154; found 569.6105; $[C_{70}@Pd_2\mathbf{L^4}_2\mathbf{L^5}(MeCN)_2]^{4+}$ (major species) calcd. for $C_{208}H_{90}N_{16}O_{12}Pd_2$ 804.3764; found 804.3694; $[C_{70}@Pd_2\mathbf{L^4}\mathbf{L^5}_3]^{4+}$ calcd. for $C_{232}H_{102}N_{22}O_{16}Pd_2$ 916.3996; found 916.3915; $[C_{70}@Pd_2\mathbf{L^4}_2\mathbf{L^5}_2]^{4+}$ calcd. for $C_{242}H_{108}N_{20}O_{16}Pd_2$ 940.9098; found 940.9014.

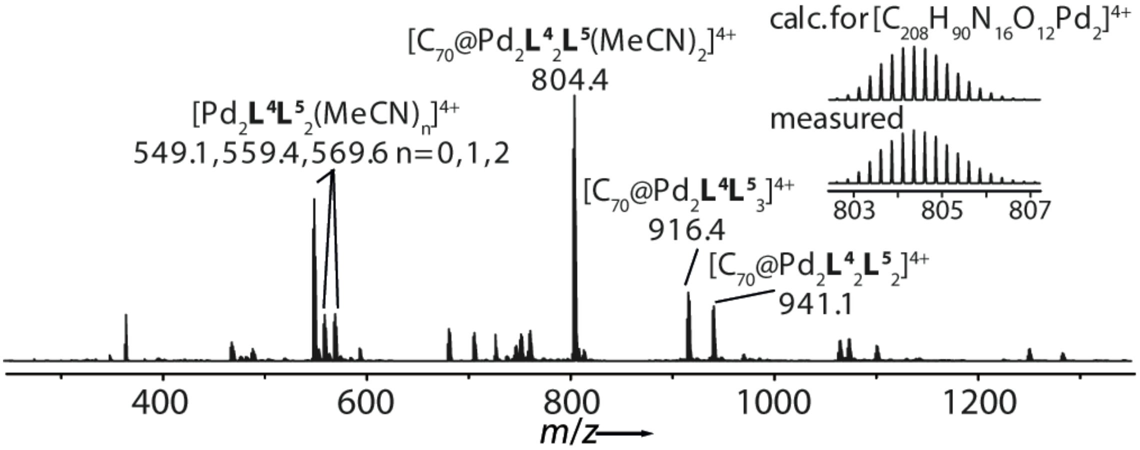

Figure S32 ESI mass spectrum of $[C_{70}@Pd_2\mathbf{L^4}_2\mathbf{L^5}(MeCN)_2]^{4+}$. The highest signal at m/z=804.4 corresponds to the major species $[C_{70}@Pd_2\mathbf{L^4}_2\mathbf{L^5}(MeCN)_2]^{4+}$, which coexists with other species.

4 Fullerene binding investigation of cage $[Pd_2L^5_4]^{4+}$

To a CD_3CN solution of $[Pd(MeCN)_4](BF_4)_2$ (1.28 mM, 1 eq.) in combination with a stoichiometric amount of ligand $\mathbf{L^5}$ (2 eq.) in a sealed vessel, excess fullerene (C_{60} or C_{70}) was added as finely grounded powders, respectively. The mixtures were sonicated for 3 minutes, then stirred at room temperature or left standing at 70 °C for few days. Upon cooling, the supernatant was collected and transferred to NMR tubes.

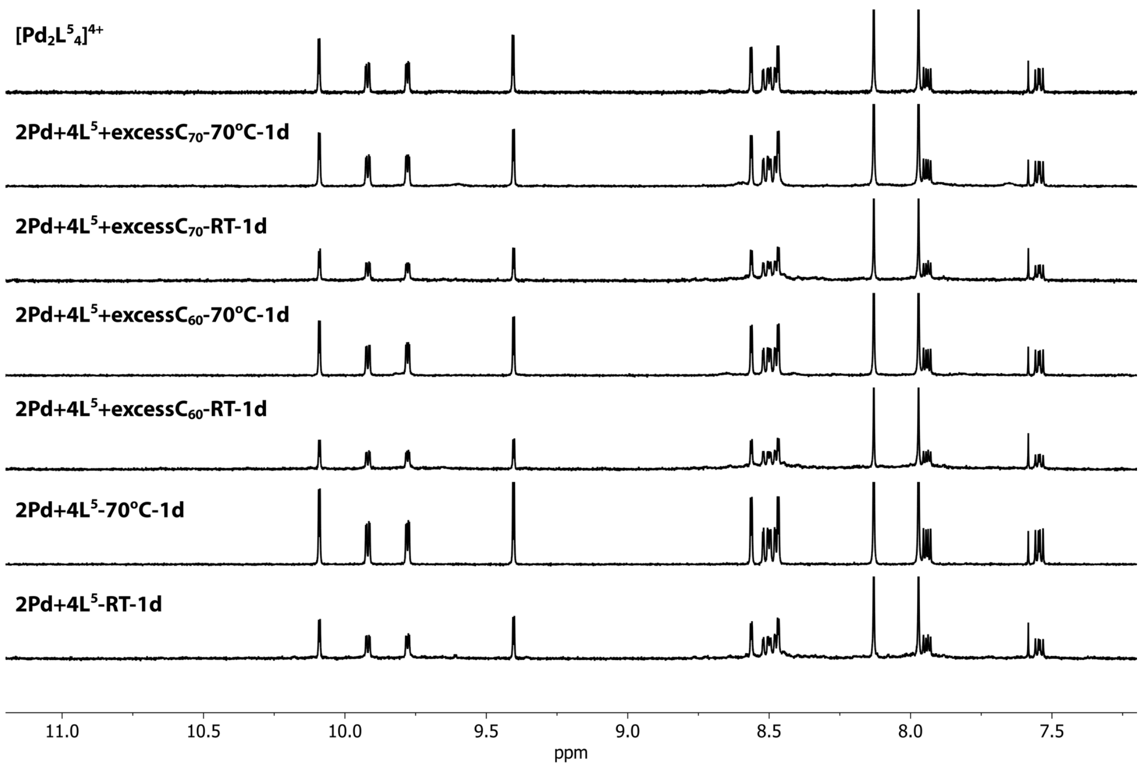

Figure S33 ^{1}H NMR spectra (500 MHz, 298 K, CD_3CN) monitoring the test of binding C_{60} and C_{70} within $[Pd_2\mathbf{L^5}_4]^{4+}$ at room temperature or 70 °C, indicating both C_{60} and C_{70} cannot be encapsulated in $[Pd_2\mathbf{L^5}_4]^{4+}$. Besides, no further change in NMR spectra was observed after prolonged heating.

5 Reactions of mixed ligand systems

5.1 Reactions of $[Pd(MeCN)_4](BF_4)_2$, ligands L^2 and L^5

The CH_2Cl_2 solution of $\mathbf{L^2}$ (5 mM) and the CH_2Cl_2 solution of $\mathbf{L^5}$ (5 mM) were respectively added to NMR tubes according to the amounts shown in the table below, followed by slowly evaporating CH_2Cl_2 from NMR tubes under heating. After sequentially adding the corresponding amounts of CD_3CN and a concentrated solution of $[Pd(MeCN)_4](BF_4)_2$ (CD_3CN, 15 mM) as well as excess fullerene powder, the mixtures were sonicated for 3 minutes and then left standing at 70 °C for several days. Upon cooling, ^{1}H NMR spectra were recorded immediately for each sample.

Table S2 Details about reaction of $[Pd(MeCN)_4](BF_4)_2$, ligands **L²** and **L⁵**.

Entries	Different amounts of reactants					
	Ratios of Pd^{II}/**L²**/**L⁵**	Pd^{II} (15 mM)	**L²** (5 mM)	**L⁵** (5 mM)	CD_3CN	Excess Fullerenes
1	2:3:1	42.7 µL	192.1 µL	64.0 µL	457.3 µL	-
2	2:2:2	42.7 µL	128.0 µL	128.0 µL	457.3 µL	-
3	2:1:3	42.7 µL	64.0 µL	192.1 µL	457.3 µL	-
4	2:3:1	42.7 µL	192.1 µL	64.0 µL	457.3 µL	C_{60}: 1.5 mg
5	2:2:2	42.7 µL	128.0 µL	128.0 µL	457.3 µL	C_{60}: 1.2 mg
6	2:1:3	42.7 µL	64.0 µL	192.1 µL	457.3 µL	C_{60}: 1.4 mg
7	2:3:1	42.7 µL	192.1 µL	64.0 µL	457.3 µL	C_{70}: 1.1 mg
8	2:2:2	42.7 µL	128.0 µL	128.0 µL	457.3 µL	C_{70}: 2.3 mg
9	2:1:3	42.7 µL	64.0 µL	192.1 µL	457.3 µL	C_{70}: 1.4 mg

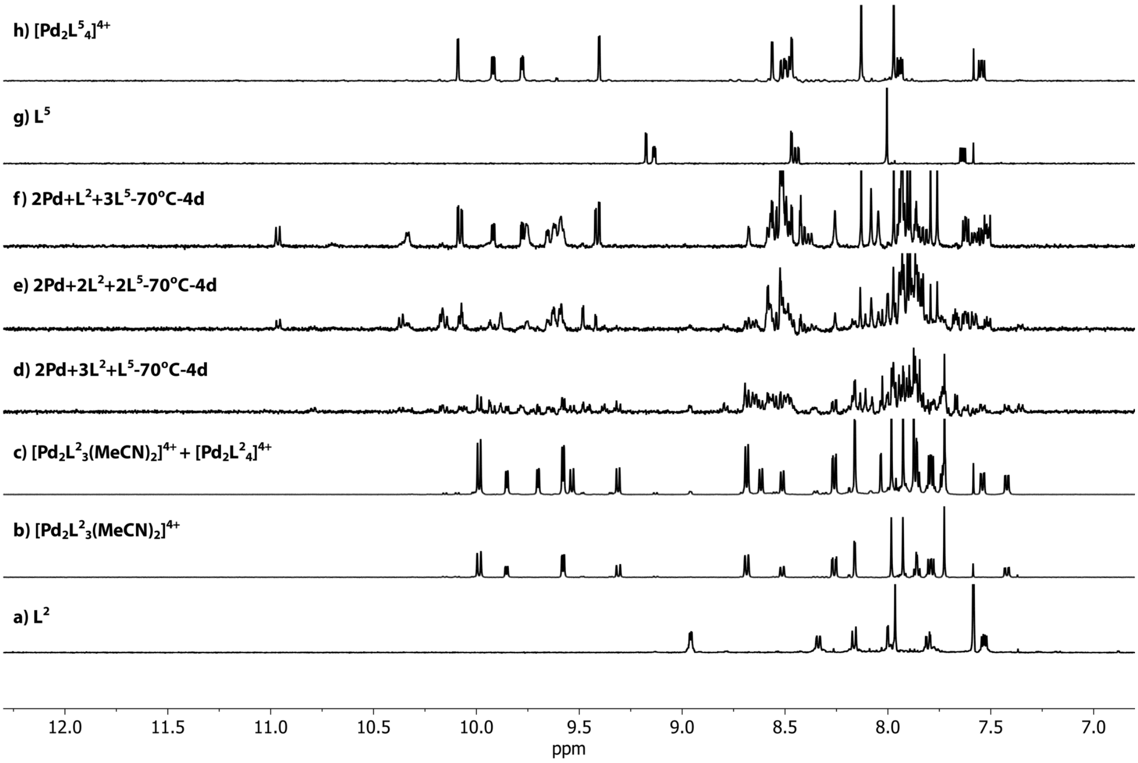

Figure S34 ^1H NMR spectra (500 MHz, 298 K, CD_3CN): (a) Ligand **L²**; (b) bowl $[Pd_2$**L²**$_3(MeCN)_2]^{4+}$; (c) the mixture of bowl $[Pd_2$**L²**$_3(MeCN)_2]^{4+}$ and cage $[Pd_2$**L²**$_4]^{4+}$; (d) reaction of Pd^{II}/**L²**/**L⁵** in a 2:3:1 ratio gave a convoluted mixture (Entry 1); (e) reaction of Pd^{II}/**L²**/**L⁵** in a 2:2:2 ratio gave a convoluted mixture (Entry 2); (f) reaction of Pd^{II}/**L²**/**L⁵** in a 2:1:3 ratio gave a convoluted mixture, including cage $[Pd_2$**L⁵**$_4]^{4+}$ (Entry 3); (g) ligand **L⁵**; (h) cage $[Pd_2$**L⁵**$_4]^{4+}$.

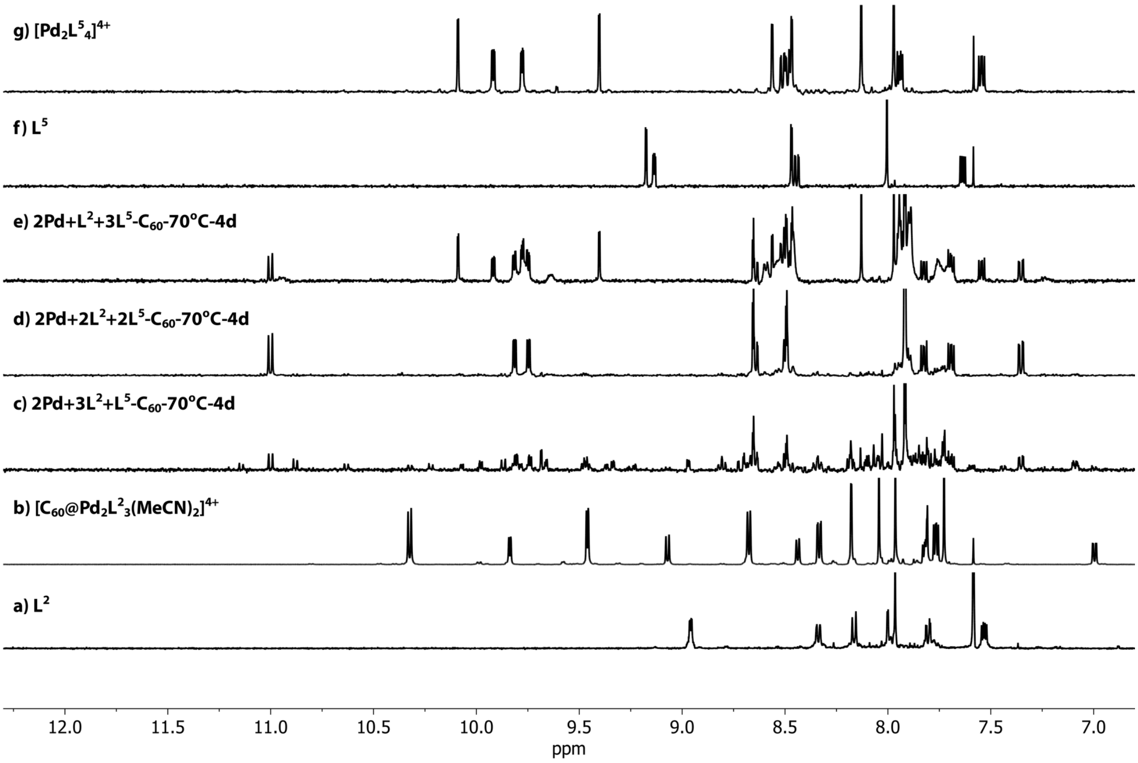

Figure S35 ^{1}H NMR spectra (500 MHz, 298 K, CD_3CN): (a) Ligand **L²**; (b) bowl $[C_{60}@Pd_2\mathbf{L^2}_3(MeCN)_2]^{4+}$; (c) reaction of Pd^{II}/**L²**/**L⁵** in a 2:3:1 ratio with excess C_{60} gave an unknown mixture (Entry 4); (d) reaction of Pd^{II}/**L²**/**L⁵** in a 2:2:2 ratio with excess C_{60} gave a well-defined spectrum, identified as cage $[C_{60}@Pd_2\mathbf{L^2}_2\mathbf{L^5}_2]^{4+}$ (Entry 5); (e) reaction of Pd^{II}/**L²**/**L⁵** in a 2:1:3 ratio with excess C_{60} gave a mixture of cage $[C_{60}@Pd_2\mathbf{L^2}_2\mathbf{L^5}_2]^{4+}$ and cage $[Pd_2\mathbf{L^5}_4]^{4+}$ (Entry 6); (f) ligand **L⁵**; (g) cage $[Pd_2\mathbf{L^5}_4]^{4+}$.

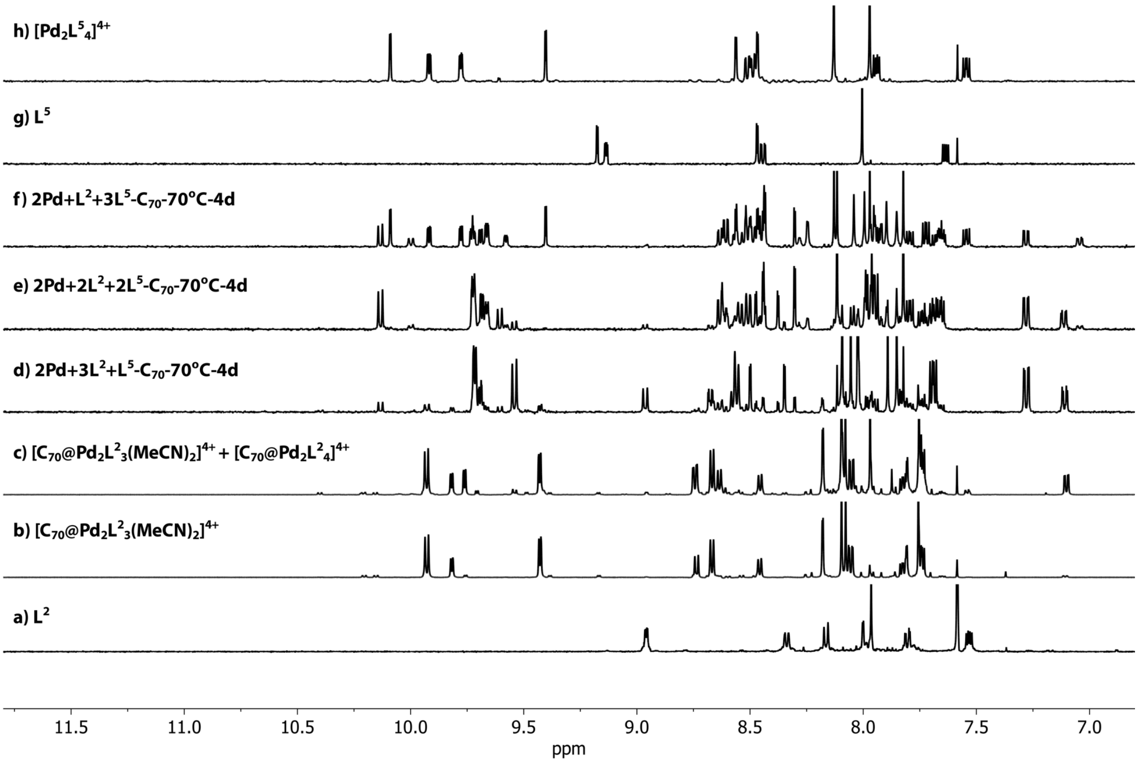

Figure S36 ^{1}H NMR spectra (500 MHz, 298 K, CD_3CN): (a) Ligand **L²**; (b) bowl $[C_{70}@Pd_2\mathbf{L^2}_3(MeCN)_2]^{4+}$; (c) the mixture of bowl $[C_{70}@Pd_2\mathbf{L^2}_3(MeCN)_2]^{4+}$ and cage $[C_{70}@Pd_2\mathbf{L^2}_4]^{4+}$; (d) reaction of Pd^{II}/**L²**/**L⁵** in a 2:3:1 ratio with excess C_{70} gave a well-defined spectrum, identified as cage $[C_{70}@Pd_2\mathbf{L^2}_3\mathbf{L^5}]^{4+}$ (major species) (Entry 7); (e) reaction of Pd^{II}/**L²**/**L⁵** in a 2:2:2 ratio with excess C_{70} gave a convoluted mixture, including cage $[C_{70}@Pd_2\mathbf{L^2}_2\mathbf{L^5}_2]^{4+}$ and cage $[C_{70}@Pd_2\mathbf{L^2}_3\mathbf{L^5}]^{4+}$ (Entry 8); (f) reaction of Pd^{II}/**L²**/**L⁵** in a 2:1:3 ratio with excess C_{70} gave a convoluted mixture, including cage $[Pd_2\mathbf{L^5}_4]^{4+}$ and cage $[C_{70}@Pd_2\mathbf{L^2}_2\mathbf{L^5}_2]^{4+}$ (Entry 9); (g) ligand **L⁵**; (h) cage $[Pd_2\mathbf{L^5}_4]^{4+}$.

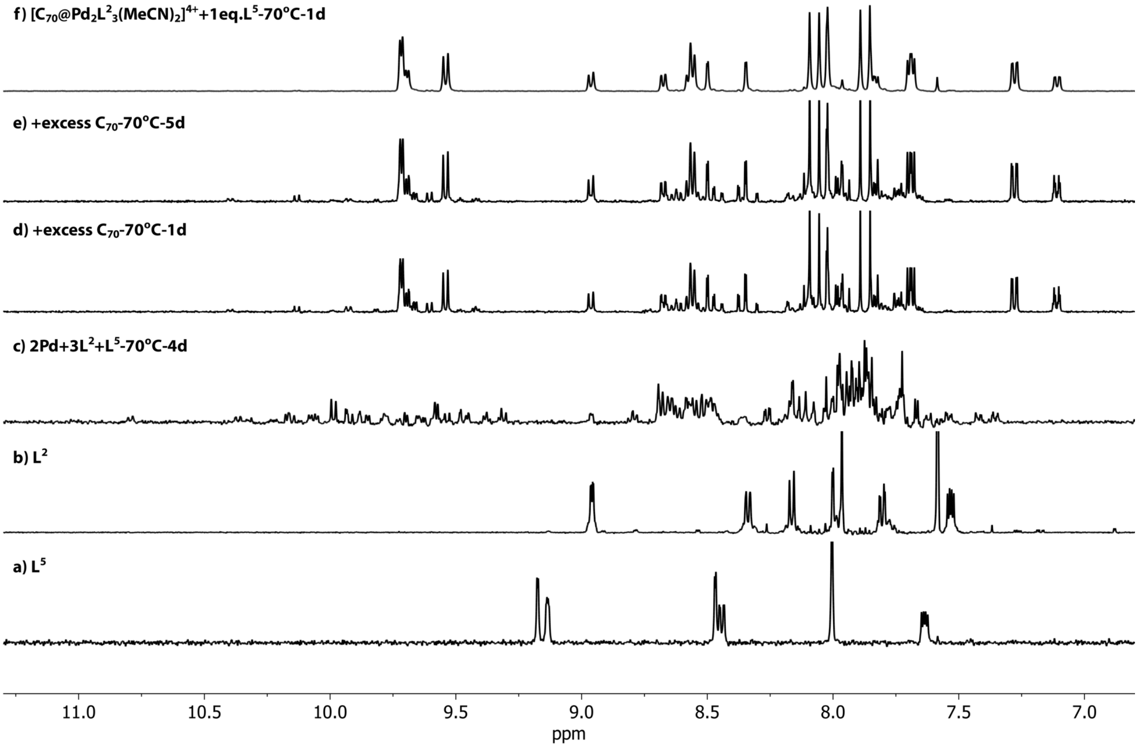

Figure S37 ^{1}H NMR spectra (500 MHz, 298 K, CD_3CN): (a) Ligand **L⁵**; (b) ligand **L²**; (b) bowl $[C_{70}@Pd_2\mathbf{L^2}_3(MeCN)_2]^{4+}$; (c) reaction of Pd^{II}/**L²**/**L⁵** in a 2:3:1 ratio gave a convoluted mixture (Entry 1); (d) and (e) subsequent addition of excess C_{70} powder into the solution (c) gave well-defined spectra after one or five days, respectively; (f) cage $[C_{70}@Pd_2\mathbf{L^2}_3\mathbf{L^5}]^{4+}$ achieved through the reaction of bowl $[C_{70}@Pd_2\mathbf{L^2}_3(MeCN)_2]^{4+}$ with 1 equivalent amount of **L⁵**.

5.2 Reactions between $[Pd(MeCN)_4](BF_4)_2$, ligands L^4 and L^5

The CH_2Cl_2 solution of $\mathbf{L^4}$ (5 mM) and the CH_2Cl_2 solution of $\mathbf{L^5}$ (5 mM) were respectively added to NMR tubes according to the amounts as shown in the table below, followed by slowly evaporating CH_2Cl_2 from NMR tubes under heating. After sequentially adding the corresponding amounts of CD_3CN and a concentrated solution of $[Pd(MeCN)_4](BF_4)_2$ (CD_3CN, 15 mM) as well as excess fullerene powder, the mixtures were sonicated for 3 minutes and then left standing at 70 °C for several days. Upon cooling, ^{1}H NMR spectra were recorded immediately for each sample.

Table S3 Details about reactions of $[Pd(MeCN)_4](BF_4)_2$, ligands **L⁴** and **L⁵**.

Entries	Different amounts of reactants					
	Ratios of Pd^{II}/**L⁴**/**L⁵**	Pd^{II} (15 mM)	**L⁴** (5 mM)	**L⁵** (5 mM)	CD_3CN	Excess Fullerenes
10	2:3:1	56.0 µL	252 µL	84 µL	600 µL	-
11	2:3:1	56.0 µL	252 µL	84 µL	600 µL	C_{60}: 1.2 mg
12	2:3:1	56.0 µL	252 µL	84 µL	600 µL	C_{70}: 1.1 mg
13	2:2:2	56.0 µL	168 µL	168 µL	600 µL	-
14	2:2:2	56.0 µL	168 µL	168 µL	600 µL	C_{60}: 2.3 mg
15	2:2:2	56.0 µL	168 µL	168 µL	600 µL	C_{70}: 0.8 mg
16	2:1:3	56.0 µL	84 µL	252 µL	600 µL	-
17	2:1:3	56.0 µL	84 µL	252 µL	600 µL	C_{60}: 1.2 mg
18	2:1:3	56.0 µL	84 µL	252 µL	600 µL	C_{70}: 1.8 mg

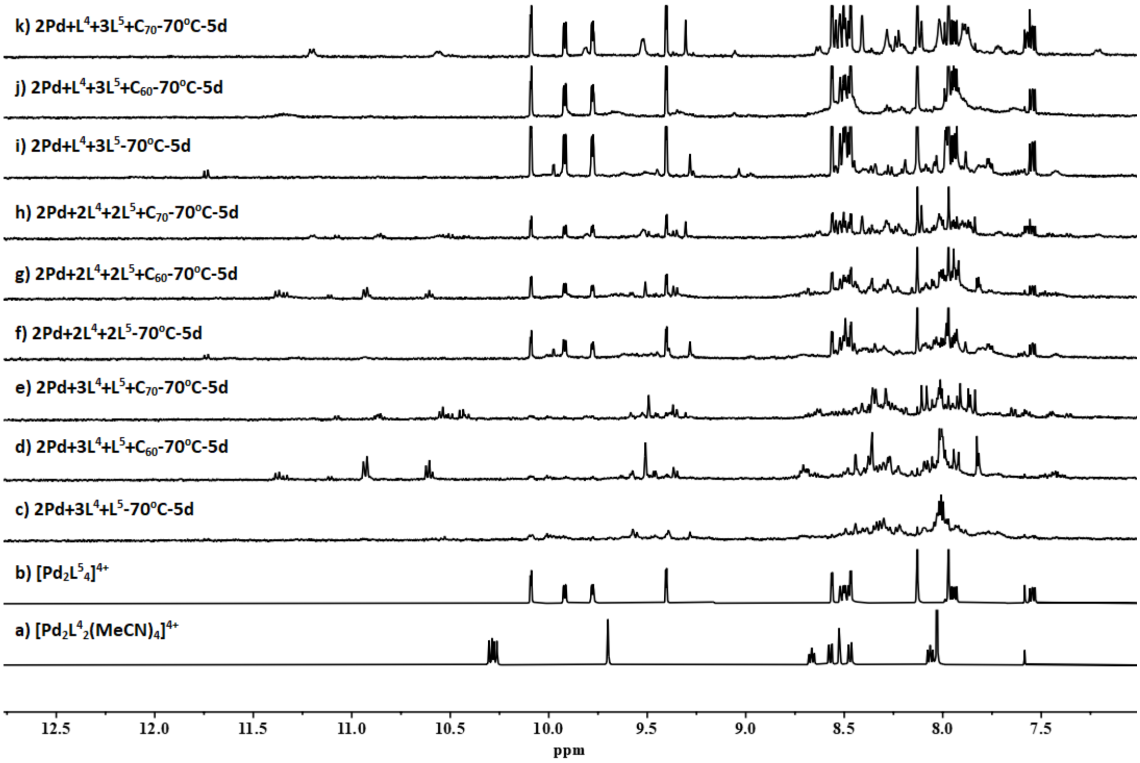

Figure S38 ^1H NMR spectra (500 MHz, 298 K, CD_3CN): (a) Ring $[Pd_2$**L⁴**$_2(MeCN)_4]^{4+}$; (b) cage $[Pd_2$**L⁵**$_4]^{4+}$; (c)-(e) reaction of Pd^{II}/**L⁴**/**L⁵** in a 2:3:1 ratio with or without fullerenes (Entries 10-12); (f)-(h) reaction of Pd^{II}/**L⁴**/**L⁵** in a 2:2:2 ratio with or without fullerenes (Entries 13-15); (i)-(k) reaction of Pd^{II}/**L⁴**/**L⁵** in a 2:1:3 ratio with or without fullerenes (Entries 16-18).

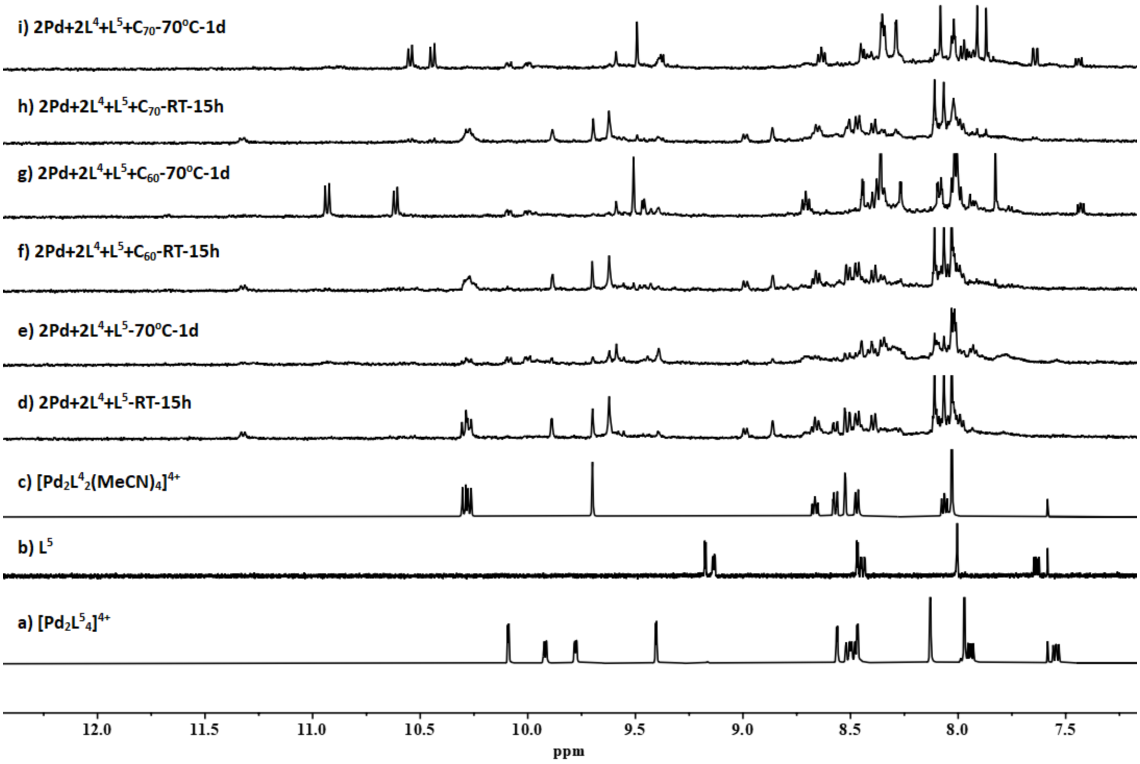

Figure S39 ^{1}H NMR spectra (500 MHz, 298 K, CD_3CN): (a) Cage $[Pd_2\mathbf{L^5}_4]^{4+}$; (b) ligand $\mathbf{L^5}$; (c) ring $[Pd_2\mathbf{L^4}_2(MeCN)_4]^{4+}$; (d) and (e) reaction of Pd^{II}/$\mathbf{L^4}$/$\mathbf{L^5}$ in a 2:2:1 ratio at room temperature and 70 °C, respectively; (f) and (g) reaction of Pd^{II}/$\mathbf{L^4}$/$\mathbf{L^5}$ in a 2:2:1 ratio in the presence of excess C_{60} at room temperature and 70 °C, respectively; (h) and (i) reaction of Pd^{II}/$\mathbf{L^4}$/$\mathbf{L^5}$ in a 2:2:1 ratio in the presence of excess C_{70} at room temperature and 70 °C, respectively.

6 X-Ray data

6.1 General methods

Ligand $\mathbf{L^5}$ and its supramolecular assemblies $[Pd_2\mathbf{L^5}_4](SbF_6)_4$, $[C_{70}@Pd_2\mathbf{L^4}_2\mathbf{L^5}(OAc)_2](BF_4)_2(C_6H_6)_2$ were studied using single crystal X-ray crystallography. The crystals of $[Pd_2\mathbf{L^5}_4](SbF_6)_4$ were extremely sensitive to loss of organic solvent. Due to very thin (5 µm) needle-like crystals the analysis was further hampered by the limited scattering power of the samples not allowing to reach the desired (sub-)atomic resolution using our a modern microfocussed X-ray in-house CuK_α source. Gaining detailed structural insight thus required cryogenic crystal handling and highly brilliant synchrotron radiation. Hence, diffraction data of $[Pd_2\mathbf{L^5}_4](SbF_6)_4$ and $[C_{70}@Pd_2\mathbf{L^4}_2\mathbf{L^5}(OAc)_2](BF_4)_2(C_6H_6)_2$ was collected at macromolecular synchrotron beamline P11, PETRA III, DESY.[4] Modelling of counterion required carefully adapted macromolecular refinement protocols employing geometrical restraint dictionaries, similarity restraints and restraints for anisotropic displacement parameters (ADPs).

Table S4 Crystallographic data of **L**^{5}, [Pd_2**L**$^5_4$](SbF_6)$_4$ and [C_{70}@Pd_2**L**$^4_2$**L**^{5}(OAc)$_2$](BF_4)$_2$(C_6H_6)$_2$.

Compound	**L**^{5}	**[$Pd_2L^5_4$](SbF_6)$_4$**	**[C_{70}@$Pd_2L^4_2L^5$(OAc)$_2$](BF_4)$_2$(C_6H_6)$_2$**
CCDC number	1997307	1997308	1997309
Identification code	bc18a	bc24b2_sq	bc31a_sq
Empirical formula	$C_{38}H_{24}N_6O_4$	$C_{608}H_{384}N_{96}O_{64}Pd_8Sb_{10}F_{60}$	$C_{220}H_{102}N_{14}O_{16}Pd_2B_2F_8$
Formula weight	628.63	13266.79	3583.57
Temperature (K)	100(2)	80(2)	100(2)
Crystal system	Monoclinic	Orthorhombic	Monoclinic
Space group	$P2_1/c$	*P*ccn	$P2_1/c$
a (Å)	14.0425(5)	41.972(8)	30.755(6)
b (Å)	11.2236(4)	70.711(14)	16.741(3)
c (Å)	19.5237(7)	27.616(6)	41.053(8)
α (º)	90	90	90
β (º)	103.069(2)	90	108.20(3)
γ (º)	90	90	90
Volume (Å^3)	2997.38(19)	81961(28)	20080(8)
Z	4	4	4
Density (calc.) (g/cm^3)	1.393	1.075	1.185
Absorption coefficient (mm^{-1})	0.093	0.511	0.661
F(000)	1304	26536	7288
Crystal size (mm^3)	0.500 x 0.020 x 0.020	0.570 x 0.003 x 0.003	0.150×0.020×0.005
θ range for data collection (º)	2.107 to 36.380	0.547 to 15.734	1.013 to 24.834
Reflections collected	90407	97809	68636
Observed reflections [R(int)]	14541 [0.0672]	20884 [0.0936]	11207 [0.0949]
Goodness-of-fit on F^2	1.017	1.649	1.665
R_1 [$I>2\sigma(I)$]	0.0493	0.1294	0.1188
wR_2 (all data)	0.1387	0.4033	0.3706
Largest diff. peak and hole (e.Å^{-3})	0.53 and -0.29	2.04 and -1.72	0.73 and -0.64
Data / restraints / parameters	14541 / 0 / 435	20884 / 9789 / 4304	11207/30712/2990

6.2 Crystal structure of ligand L^5

Colorless needle-shaped crystals of **L**^{5} were obtained by slow evaporation of a 1.00 mM CH_3CN/CH_2Cl_2 (v/v: 1:1) solution of **L**^{5}. A single crystal in mother liquor was pipetted onto a glass slide containing NVH oil. Single crystal X-ray diffraction data was collected on a Bruker D8 venture equipped with an Incoatec microfocus source (Iµs 2.0) using Mokα radiation on a four axis κ-goniometer, equipped with an Oxford Cryostream 800 and a Photon 100 detector. Data integration was

done with SAINT. Data scaling and absorption correction were performed with SADABS. The space group was determined using XPREP.[5] The structure was solved by intrinsic phasing/direct methods using SHELXT[6] and refined with SHELXL[7] for full-matrix least-squares routines on F^2 and ShelXle[8] as a graphical user interface.

6.2.1 Specific refinement details of ligand L^5

All displacements for non-hydrogen atoms were refined anisotropically.

6.2.2 Description of the structure of ligand L^5

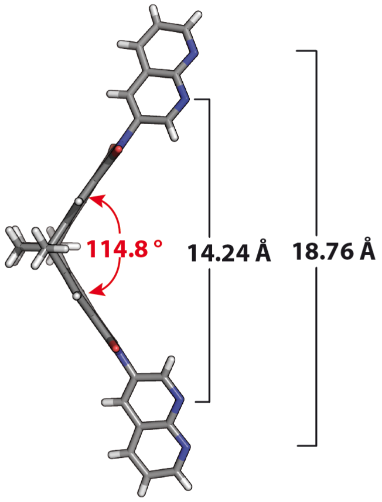

Figure S40 X-ray structure of $\mathbf{L^5}$ with the longest and shortest N–N distance of 18.76 and 14.24 Å, respectively. Color scheme: H, light grey; C, dark grey; N, blue; O, red.

6.3 Crystal structure of $[Pd_2L^5_4](SbF_6)_4$

Colorless needle-shaped crystals of $[Pd_2\mathbf{L^5}_4](SbF_6)_4$ were obtained by slow vapor diffusion of isopropyl ether into a 0.64 mM CD_3CN solution of $[Pd_2\mathbf{L^5}_4](BF_4)_4$ containing 10 eq. of $KSbF_6$. A single crystal in mother liquor was pipetted onto a glass slide containing NVH oil. To avoid collapse of the crystal lattice, the crystal was quickly mounted onto a 0.5 mm nylon loop and immediately flash cooled in liquid nitrogen. Crystals were stored at cryogenic temperature in dry shippers, in which they were safely transported to macromolecular beamline P11 at Petra III,[4] DESY, Germany.

A wavelength of λ = 0.6888 Å was chosen using a liquid N_2 cooled double crystal monochromator. Single crystal X-ray diffraction data was collected at 80(2) K on a single axis goniometer, equipped with an Oxford Cryostream 800 and a Pilatus 6M detector. 1900 diffraction images were collected in a 360° ϕ sweep at a detector distance of 200 mm, 100% filter transmission, 0.2° step width and 0.06 seconds exposure time per image. And 1650 diffraction images were used for data integration. Data integration and reduction were undertaken using XDS.[9] The structure was solved by intrinsic phasing/direct methods using SHELXT[6] and refined with SHELXL[7] using 22 cpu cores for full-matrix least-squares routines on F^2 and ShelXle[8] as a graphical user interface and the DSR program plugin was employed for modeling.[10]

6.3.1 Specific refinement details of $[Pd_2L^5{}_4](SbF_6)_6$

Stereochemical restraints for the EAN ligands (**L^5**) were generated by the GRADE program using the GRADE Web Server (http://grade.globalphasing.org) and applied in the refinement. A GRADE dictionary for SHELXL contains target values and standard deviations for 1,2-distances (DFIX) and 1,3-distances (DANG), as well as restraints for planar groups (FLAT). All displacements for non-hydrogen atoms were refined anisotropically. The refinement of ADP's for carbon, nitrogen and oxygen atoms was enabled by a combination of similarity restraints (SIMU) and rigid bond restraints (RIGU).[11] The contribution of the electron density from disordered counterions and solvent molecules, which could not be modeled with discrete atomic positions were handled using the SQUEEZE[12] routine in PLATON.[13] The solvent mask file (.fab) computed by PLATON were included in the SHELXL refinement via the ABIN instruction leaving the measured intensities untouched.

Table S5 Definition of residues involved in $[Pd_2\mathbf{L^5}_4](SbF_6)_4$.

Fragment	Residue class	Occurrence	Residue numbers
Pd^{2+}	PD	1	1
Ligand **L^5**	EAN	9	2-8, 16 (Two EAN with 50% occupation)
SbF_6^-	SBF	6	10-15 (Two SBF with 50% occupation)

6.3.2 Description of the structure of $[Pd_2L^3_4](BF_4)_4$

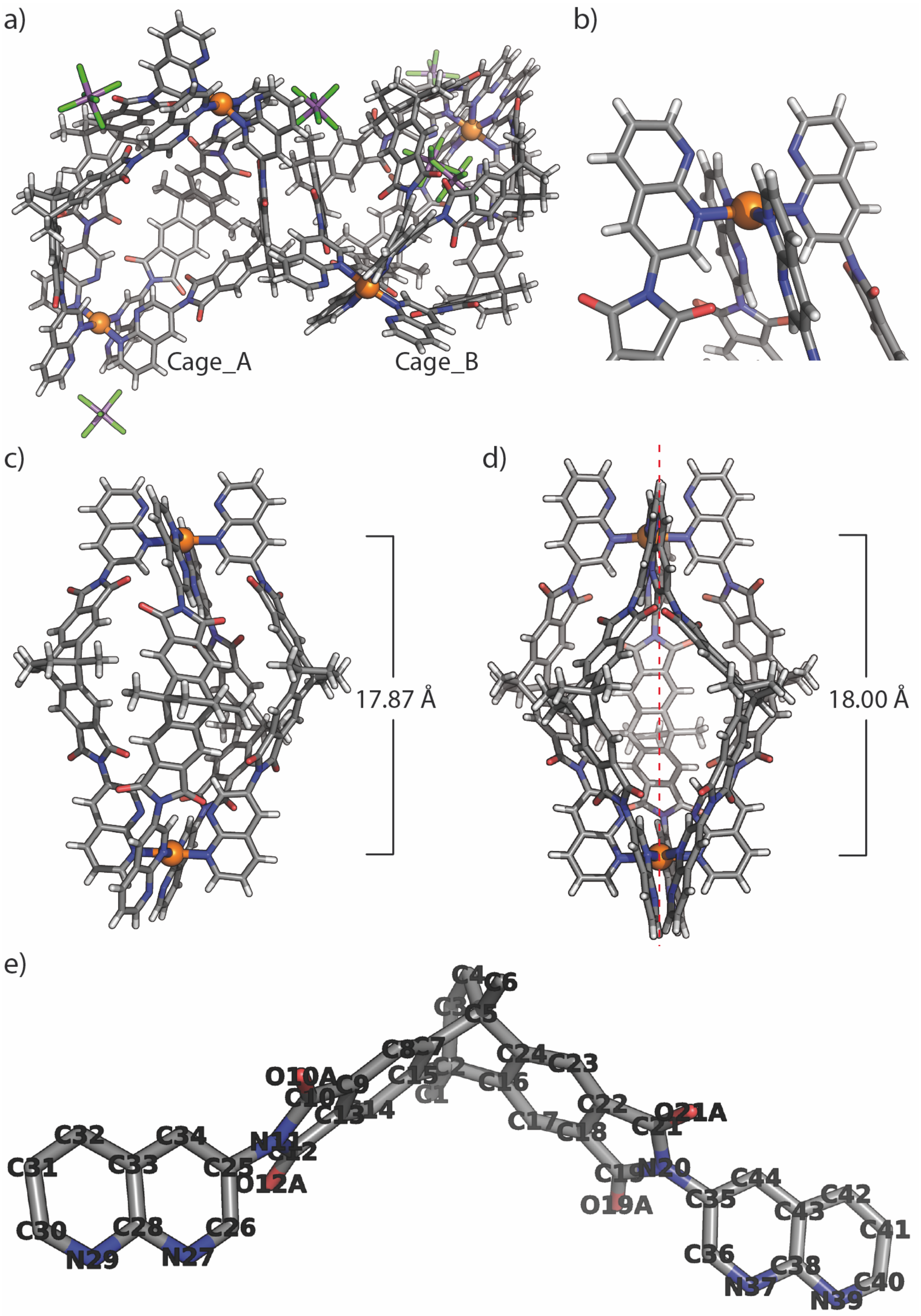

Figure S41 X-ray structure of $[Pd_2\mathbf{L^5}_4](SbF_6)_4$: (a) the asymmetric unit of two crystallographically independent cages; (b) coordination center showing the dislocation of coordinated donors; (c) cage_A showing a Pd–Pd distance of 17.87 Å; (d) cage_B showing a Pd–Pd distance of 18.00 Å and one of the ligands in two positions with 50% occupation; (e) atomic naming scheme of ligand $\mathbf{L^5}$ (residue class EAN). Color scheme: H, light grey; C, dark grey; N, blue; O, red; F, green; Pd, orange; Sb, purple.

6.4 Crystal structure of [C_{70}@Pd_2**L**$^4_2$**L**^{5}(OAc)$_2$](BF_4)$_2$(C_6H_6)$_2$

Red needle-shaped crystals of [C_{70}@Pd_2**L**$^4_2$**L**^{5}(OAc)$_2$](BF_4)$_2$(C_6H_6)$_2$ were obtained by slow vapor diffusion of benzene into a 0.64 mM CD_3CN solution of [C_{70}@Pd_2**L**$^4_2$**L**^{5}(MeCN)$_2$](BF_4)$_4$. A single crystal in mother liquor was pipetted onto a glass slide containing NVH oil. To avoid collapse of the crystal lattice, the crystal was quickly mounted onto a 0.5 mm nylon loop and immediately flash cooled in liquid nitrogen. Crystals were stored at cryogenic temperature in dry shippers, in which they were safely transported to macromolecular beamline P11 at Petra III,[4] DESY, Germany.

A wavelength of λ = 1.0332 Å was chosen using a liquid N_2 cooled double crystal monochromator. Single crystal X-ray diffraction data was collected at 100(2) K on a single axis goniometer, equipped with an Oxford Cryostream 800 and a Pilatus 6M detector. 3600 diffraction images were collected in a 360° ϕ sweep at a detector distance of 154 mm, 82.3 % filter transmission, 0.1° step width and 0.1 seconds exposure time per image. Data integration and reduction were undertaken using XDS.[9] The structure was solved by intrinsic phasing/direct methods using SHELXT[6] and refined with SHELXL[7] using 22 cpu cores for full-matrix least-squares routines on F^2 and ShelXle[8] as a graphical user interface and the DSR program plugin was employed for modeling.[10]

6.4.1 Specific refinement details of [C_{70}@Pd_2**L**$^4_2$**L**^{5}(OAc)$_2$](BF_4)$_2$(C_6H_6)$_2$

The OAc^- anions were assigned crystallographically by the electron density, although OAc^- anions were not meant to be contained in the solution of sample [C_{70}@Pd_2**L**$^4_2$**L**^{5}](BF_4)$_4$. We presume that the observed OAc^- ions came from trace impurities of the used salts or solvents, and they substituted the weakly bound MeCN during the process of crystallization. Stereochemical restraints for the EAA (**L**^{4}) and EAN ligands (**L**^{5}) were generated by the GRADE program using the GRADE Web Server (http://grade.globalphasing.org) and applied in the refinement. A GRADE dictionary for SHELXL contains target values and standard deviations for 1,2-distances (DFIX) and 1,3-distances (DANG), as well as restraints for planar groups (FLAT). All displacements for non-hydrogen atoms were refined anisotropically. The refinement of ADP's for carbon, nitrogen and oxygen atoms was enabled by a combination of similarity restraints (SIMU) and rigid bond restraints (RIGU).[11] The contribution of the electron density from disordered counterions and solvent molecules, which could not be modeled with discrete atomic positions were handled using the SQUEEZE[12] routine in PLATON.[13] The solvent mask file (.fab) computed by PLATON were included in the SHELXL refinement via the ABIN instruction leaving the measured intensities untouched.

Table S6 Definition of residues involved in [C_{70}@Pd_2**L**$^4_2$**L**^{5}(OAc)$_2$](BF_4)$_2$(C_6H_6)$_2$.

Fragment	Residue class	Occurrence	Residue numbers
Pd^{2+}	PD	1	1
Ligand **L**^{4}	EAA	2	2, 3
Ligand **L**^{5}	EAN	1	4
C_{70}	C70	2	5, 12 (Two C_{70} with 50% occupation)
OAc^-	OAC	2	6, 7
BF_4^-	BF4	2	8, 9
C_6H_6	BEN	2	10, 11

6.4.2 Description of the structure of $[C_{70}@Pd_2\mathbf{L^4}_2\mathbf{L^5}(OAc)_2](BF_4)_2(C_6H_6)_2$

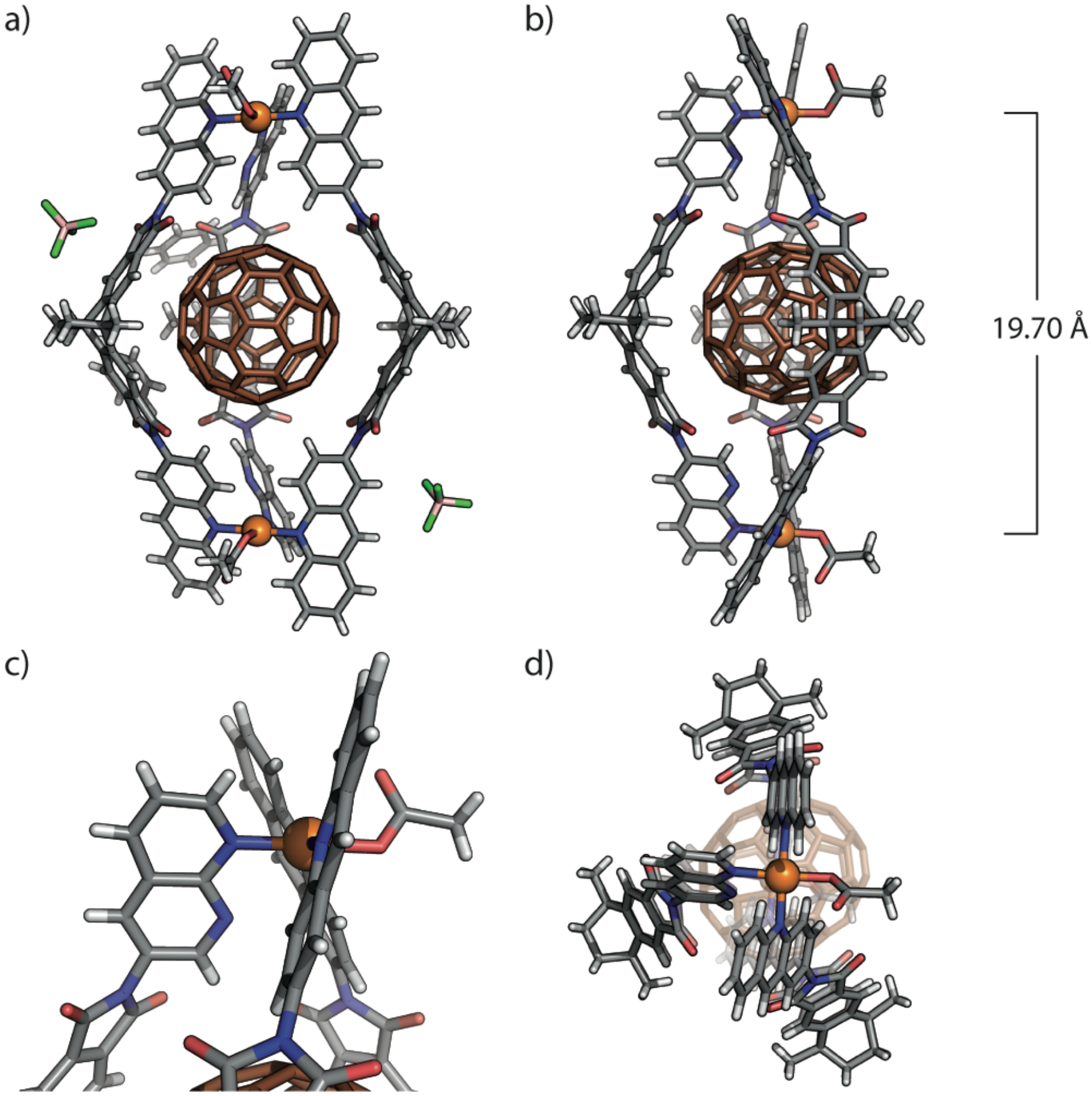

Figure S42 X-ray structure of $[C_{70}@Pd_2\mathbf{L^4}_2\mathbf{L^5}(OAc)_2](BF_4)_2(C_6H_6)_2$: (a) and (b) full structure showing a Pd–Pd distance of 19.70 A; (c) coordination center showing the combination of coordinated terminals; (d) top view. Color scheme: H, light grey; B, pink; C, dark grey; N, blue; O, red; F, green; Pd, orange; C_{70}: brown.

7 Computational studies

All models shown below were constructed using Wavefunction SPARTAN '14[14] and first optimized on semiempiric PM6 level of theory without constraints. The resulting structures were then further refined by DFT structure optimization (B3LYP/LANL2DZ) using GAUSSIAN 09.[15]

7.1 Comparison of the DFT minimized energies of A-type $[Pd_2L^5_4]^{4+}$ and B-type $[Pd_2L^5_4]^{4+}$

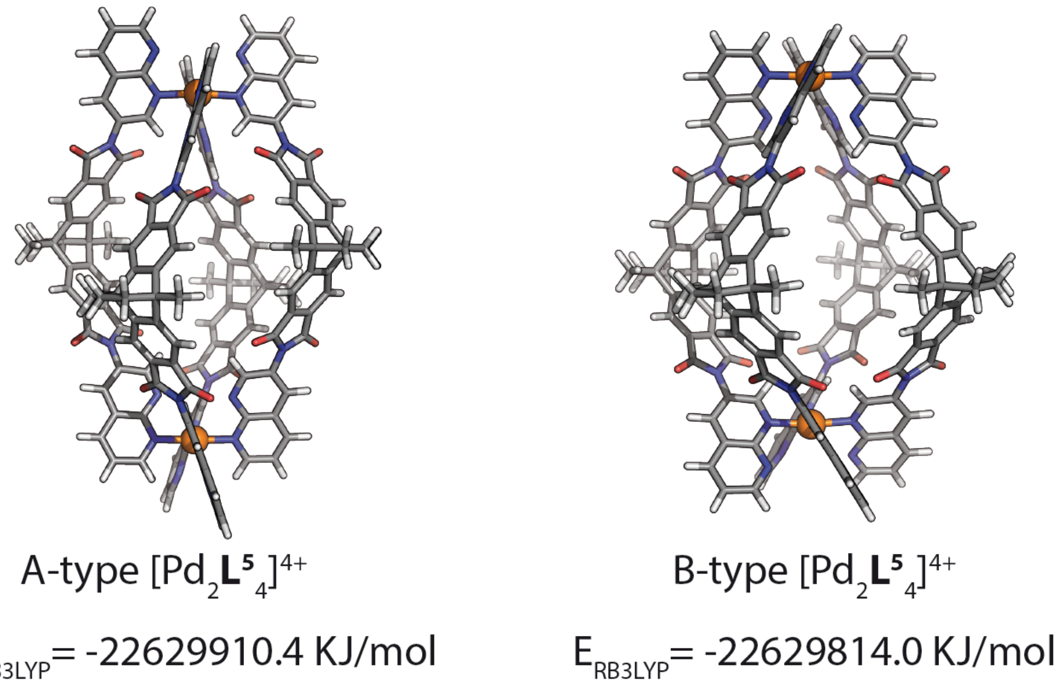

Figure S43 DFT energy minimized structures of observed A-type $[Pd_2\mathbf{L^5}_4]^{4+}$ and tentative B-type $[Pd_2\mathbf{L^5}_4]^{4+}$. According to the computed energies, A-type (dislocated) $[Pd_2\mathbf{L^5}_4]^{4+}$ is 96.4 kJ/mol lower in energy.

7.2 Comparison of the DFT minimized energies of *cis*-$[C_{60}@Pd_2L^2_2L^5_2]^{4+}$ and *trans*-$[C_{60}@Pd_2L^2_2L^5_2]^{4+}$

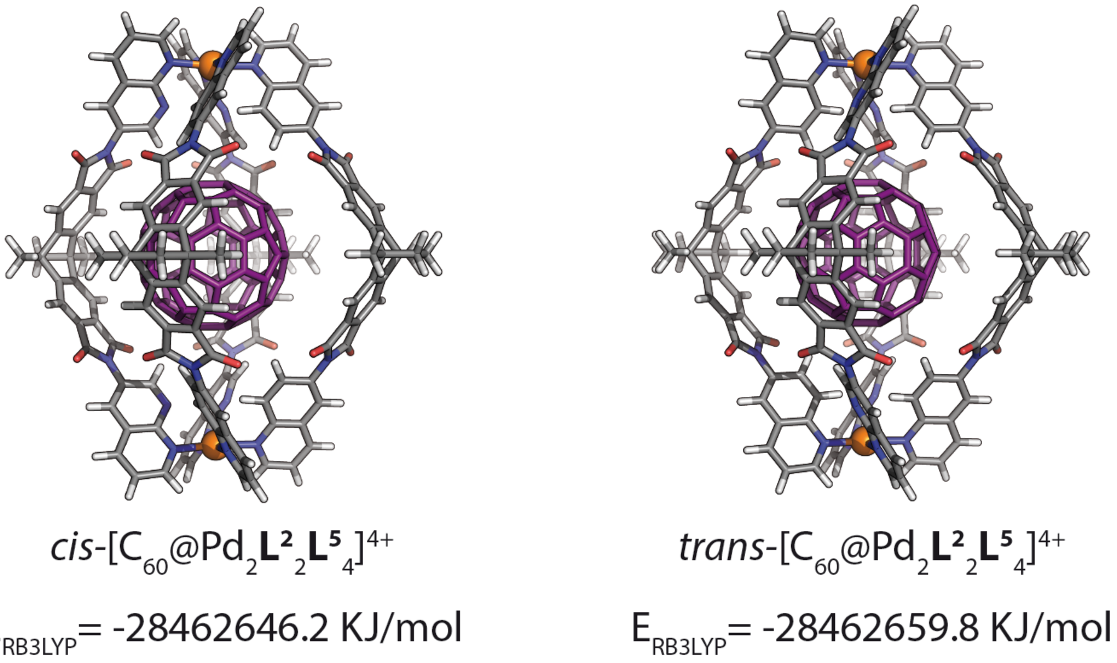

Figure S44 DFT energy minimized structures of tentative *cis*-$[C_{60}@Pd_2\mathbf{L^2}_2\mathbf{L^5}_2]^{4+}$ and observed *trans*-$[C_{60}@Pd_2\mathbf{L^2}_2\mathbf{L^5}_2]^{4+}$. According to the computed energies, *trans*-$[C_{60}@Pd_2\mathbf{L^2}_2\mathbf{L^5}_2]^{4+}$ is 13.6 kJ/mol lower in energy.

7.3 Comparison of ligand combinations in mononuclear model complexes

In order to get theoretical insight into the interaction of the naphthyridine ("N") N lone pair with naphthyridine N positions or quinoline ("Q") CH moieties in direct neighborhood among ligands coordinated to a Pd(II) center, we performed a series of calculations. First, we geometry-optimized mononuclear complex $[PdQ_4]^{2+}$ on DFT ωB97X-D/def2-SVP level in the gas phase using the Spartan software package[14] and noted its energy (–1733.526242 Hartree) as energetic reference (set to 0; following energy differences reported in kJ/mol) and the dihedral angles 43.65° between the α-C̲H next to the coordinating nitrogen, this nitrogen, the *trans*-standing nitrogen and the corresponding α-C̲H were noted and applied as constraint to all following computations (for *trans*-standing combinations of Q, N and pyridine ["Py"] ligands). Setting these constraints was necessary to obtain energies for comparable geometries (i.e. conformers) were the different functionalities (N lone pair in naphtyridine, CH hydrogen in quinoline and no substituent in the equivalent position in case of the pyridine ligands) are taking the same spatial positions, hence are not allowed to move relative to their neighbors in order to lower the overall energy by rotation around the bonds between the coordinating nitrogens and the palladium center. Such conformational freedom was not allowed for the mononuclear model complexes examined here to treat them similarly to the conformationally constrained situation found in the corresponding sub-structures of the dinuclear coordination cages.

Following ligand exchange energies were obtained:

$[PdPy_3Q]^{2+}$	+ N → $[PdPy_3N]^{2+}$ + Q	– 34.1 kJ/mol
$[PdQ_4]^{2+}$	+ N → $[PdQ_3N]^{2+}$ + Q	– 32.4 kJ/mol
$[PdQ_3N]^{2+}$	+ N → *trans*-$[PdQ_2N_2]^{2+}$ + Q	– 29.4 kJ/mol (*cis*-$[PdQ_2N_2]^{2+}$ + 8.1 kJ/mol higher than *trans*)
trans-$[PdQ_2N_2]^{2+}$	+ N → $[PdQN_3]^{2+}$ + Q	– 10.0 kJ/mol
$[PdQN_3]^{2+}$	+ N → $[PdN_4]^{2+}$ + Q	– 5.0 kJ/mol

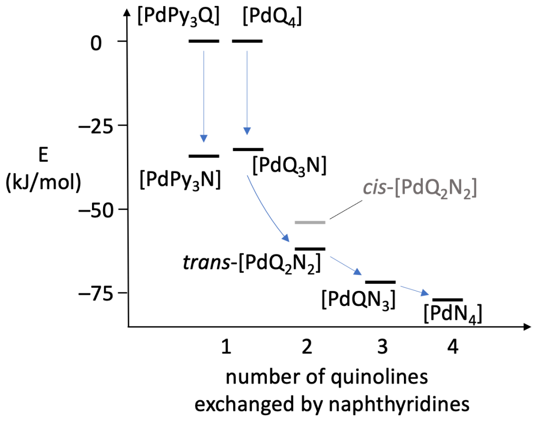

Figure 45 Energy scheme of consecutive ligand exchange (Q→N) from $[PdQ_4]^{2+}$ to $[PdN_4]^{2+}$ (and $[PdPy_3Q]^{2+}$ to $[PdPy_3N]^{2+}$ for comparison)

The calculations reveal that 1) exchange of Q by N in general goes energetically downhill and 2) the first exchange of Q for N, starting from $[PdQ_4]^{2+}$, is energetically most favorable, closely followed by a second exchange (but only for formation of the *trans*-product in which the lone pairs of N ligands are not in direct neighborhood!), while 3) the introduction of a third - and even more so - a fourth N ligand is energetically significantly less advantageous, attributed to the nitrogen lone-pair repulsion between naphthyridine ligands in direct neighborhood.

8 References

[1] B. Chen, J. J. Holstein, S. Horiuchi, W. G. Hiller, G. H. Clever, *J. Am. Chem. Soc.* **2019**, *141*, 8907-8913.
[2] B. Chen, S. Horiuchi, J. J. Holstein, J. Tessarolo, G. H. Clever, *Chem. Eur. J.* **2019**, *25*, 14921-14927.
[3] Y. Rogan, R. Malpass-Evans, M. Carta, M. Lee, J. C. Jansen, P. Bernardo, G. Clarizia, E. Tocci, K. Friess, M. Lanč, N. B. McKeown, *J. Mater. Chem. A* **2014**, *2*, 4874-4877.
[4] A. Burkhardt, T. Pakendorf, B. Reime, J. Meyer, P. Fischer, N. Stube, S. Panneerselvam, O. Lorbeer, K. Stachnik, M. Warmer, P. Rodig, D. Gories, A. Meents, *Eur. Phys. J. Plus* **2016**, *131*, 1-9.
[5] Bruker-Nonius, APEX, SAINT, SADABS and XPREP, Bruker AXS Inc., Madison (USA), **2013**.
[6] G. Sheldrick, *Acta Crystallogr. Sect. A* **2015**, *71*, 3-8.
[7] G. Sheldrick, *Acta Crystallogr. Sect. C* **2015**, *71*, 3-8.
[8] C. B. Hubschle, G. M. Sheldrick, B. Dittrich, *J. Appl. Crystallogr.* **2011**, *44*, 1281-1284.
[9] W. Kabsch, *Acta Crystallogr. Sect. D* **2010**, *66*, 125-132.
[10] D. Kratzert, J. J. Holstein, I. Krossing, *J. Appl. Crystallogr.* **2015**, *48*, 933-938.
[11] A. Thorn, B. Dittrich, G. M. Sheldrick, *Acta Crystallogr. Sect. A* **2012**, *68*, 448-451.
[12] A. Spek, *Acta Crystallogr. Sect. C* **2015**, *71*, 9-18.
[13] A. Spek, *Acta Crystallogr. Sect. D* **2009**, *65*, 148-155.
[14] Spartan ’08 Version 1.2.0, Wavefunction, Inc., Irvine (USA), **2009**.
[15] M. J. Frisch, et al., Gaussian09, Gaussian Inc., Wallingford (USA), **2009**.
